# The role of dietary preferences in osteoarthritis: a Mendelian randomization study using genome-wide association analysis data from the UK Biobank

**DOI:** 10.3389/fnut.2024.1373850

**Published:** 2024-04-29

**Authors:** Long Chen, Yiqi Su, Hui Li, Zhen Yang, Jiao Jiao Li, Dan Xing

**Affiliations:** ^1^Arthritis Clinic and Research Center, Peking University People’s Hospital, Peking University, Beijing, China; ^2^School of Biomedical Engineering, Faculty of Engineering and IT, University of Technology Sydney, Sydney, NSW, Australia

**Keywords:** osteoarthritis, dietary preferences, Mendelian randomization, causality, SNP

## Abstract

**Background:**

To understand the impact of individual preferences for specific dietary items on OA, and to help inform the development of effective and targeted OA prevention and management strategies, we performed a Mendelian randomization analysis between dietary preferences and osteoarthritis.

**Methods:**

This study utilized genetic data from the UK Biobank to investigate the association between OA and 21 different common dietary items. Instrumental variables representing European populations were carefully selected based on their genetic significance and linkage disequilibrium. In cases where a dietary item had few relevant genetic markers, a more lenient selection threshold was applied. To prevent bias, the analysis excluded single nucleotide polymorphisms (SNPs) associated with factors such as body mass index (BMI) and cholesterol. Using inverse-variance weighting (IVW) and Mendelian randomization, significant associations were detected between certain dietary items and OA.

**Results:**

Using Mendelian randomization to examine the relationship between 21 different dietary items and OA, significant associations were found for coffee, peas, watercress, and cheese, where the first two had a promoting effect and the last two an inhibiting effect on OA. Due to heterogeneity in the test results for cheese, a random IVW representation was used. The results of sensitivity analysis showed no significant heterogeneity or horizontal pleiotropy in the selected SNPS, demonstrating the reliability of Mendelian randomization analysis.

**Conclusion:**

This study identified coffee, peas, watercress, and cheese as food items that may have significant dietary effects on osteoarthritis. This information may be useful to consider in the development of OA management strategies.

## 1 Introduction

Osteoarthritis (OA) is a prevalent joint disorder characterized by clinical symptoms such as chronic pain, crepitus, joint stiffness, and structural changes including radiographic alterations and joint-wide tissue degradation (1). The socioeconomic influences of OA are globally recognized as imposing a staggering long-term burden on both individuals and healthcare systems due to the absence of a curative treatment and need for chronic management (2, 3). The pathogenesis of OA is complex, involving a variety of genetic, lifestyle, and other factors (4), and is not fully understood. Certain lifestyle and health factors are found to be definitive risk factors for OA, such as excessive physical activity, alcohol consumption, smoking, type 2 diabetes, and obesity (5). Current studies have outlined a role of diet on OA progression, although mostly centered on the association between diet and obesity and consequent effects on OA (6, 7), or specific types of diet (8).

Research into the effects of diet on OA has yielded interesting, sometimes conflicting results (6, 9–11). There is good evidence to suggest that certain dietary components such as dietary fiber, lipids, and vitamins have a significant impact on OA progression (12–14). These nutrients may influence key molecular pathways implicated in OA pathogenesis, by changing serum lipid concentrations, expression of inflammatory biomarkers, oxidative stress responses, and the activity of matrix metalloproteinases, among other mechanisms (12, 15). There is a gap in research regarding the comprehensive impact of specific foods on osteoarthritis. This study aims to explore the association between specific food items, including coffee, tea, fruits, dairies, meats, vegetables, and nuts, and osteoarthritis using genetic data from the UK Biobank.

The UK Biobank is an invaluable resource for biomedical research, providing a vast repository of detailed genetic, lifestyle, and health information from around 500,000 participants in the UK (16). This allows multifaceted analysis particularly regarding the associations and risk factors of common health problems such as OA. Of particular significance, the database contains genome-wide association analysis data for various dietary intake preferences. The IEU open GWAS database is a comprehensive platform that integrates genome-wide association study data from various sources. The data for our GWAS study on diet were sourced from the summary data of the most recent UK Biobank GWAS available in the IEU Open GWAS database. Mendelian randomization (MR) is an analytical method that conducts causal inferences by using ancestral genetic variations as instrumental variables for exposures (such as dietary preference for milk) and outcomes (such as OA) (17). It takes advantage of the fact that single nucleotide polymorphisms (SNPs) are randomly allocated at conception, avoiding issues of reverse causality and reducing residual confounding. Mendelian randomization is grounded on three key assumptions: (i) Correlation assumption: A robust correlation exists between SNP and exposure factors (ii) Independence assumption: SNP and confounding factors are independent (iii) Exclusion hypothesis: SNP can solely impact the outcome through exposure factors. In this study, we conducted a two-sample Mendelian analysis to investigate the causal relationship between specific dietary preferences and OA. We selected representative food items as exposures and used OA as the outcome. This approach should allow an accurate assessment of the impact of dietary choices on OA risk. The study findings may enhance the current understanding of how common food items affect OA, and inform the development of more effective dietary strategies for disease prevention and its chronic management, particularly in predisposing populations such as those who are overweight or have had prior joint injury.

## 2 Materials and methods

### 2.1 Selection of instrumental variables and data source

The genetic variation in dietary intake was obtained from publicly available data from the UK Biobank cohort, consisting of approximately half a million people (16). From the original list of dietary items, we selected 21 by adaptation of published methods (18), namely coffee, tea, cheese, cereal, pork, fresh fruit, dried fruit, cooked vegetable, salad/raw vegetable, bread, peas, unsalted peanuts, salted peanuts, milk, yogurt, beef, unsalted nuts, salted nuts, lamb, Indian snacks, and watercress (Table 1). The foods we selected considered a wide range of common dietary categories including drinks, dairy products, meat, vegetables, fruits, nuts, snacks, and staple foods.

**Table 1:** Results of Mendelian randomized analysis of all dietary preferences.

Exposure	Method	OR (95%OR)*	*p*
Coffee intake	MR Egger	1.281 (0.487,3.366)	0.620
	Weighted median	1.741 (1.168,2.594)	0.006
	Inverse variance weighted	1.543 (1.145,2.079)	0.004*
	Simple mode	1.541 (0.665,3.573)	0.322
	Weighted mode	1.766 (0.751,4.155)	0.204
Tea intake	MR Egger	3.5 (1.255,9.762)	0.023
	Weighted median	1.135 (0.817,1.578)	0.449
	Inverse variance weighted	1.249 (0.935,1.67)	0.132
	Simple mode	0.968 (0.448,2.093)	0.935
	Weighted mode	0.927 (0.325,2.647)	0.888
Cheese intake	MR Egger	0.669 (0.298,1.503)	0.335
	Weighted median	0.893 (0.714,1.117)	0.321
	Inverse variance weighted	0.737 (0.603,0.901)	0.003*
	Simple mode	0.987 (0.605,1.609)	0.957
	Weighted mode	0.977 (0.638,1.496)	0.917
Cereal intake	MR Egger	1.912 (0.244,14.954)	0.543
	Weighted median	0.684 (0.457,1.023)	0.064
	Inverse variance weighted	0.7 (0.489,1.002)	0.051
	Simple mode	0.648 (0.301,1.394)	0.278
	Weighted mode	0.661 (0.322,1.359)	0.271
Pork intake	MR Egger	0.362 (0.002,60.937)	0.709
	Weighted median	1.69 (0.736,3.883)	0.216
	Inverse variance weighted	1.78 (0.845,3.751)	0.129
	Simple mode	3.435 (0.844,13.975)	0.123
	Weighted mode	3.112 (0.775,12.487)	0.148
Fresh fruit intake	MR Egger	0.805 (0.257,2.525)	0.712
	Weighted median	0.859 (0.537,1.374)	0.526
	Inverse variance weighted	0.988 (0.701,1.394)	0.946
	Simple mode	0.775 (0.345,1.739)	0.541
	Weighted mode	0.816 (0.425,1.566)	0.545
Dried fruit intake	MR Egger	0.782 (0.073,8.392)	0.841
	Weighted median	0.978 (0.626,1.528)	0.922
	Inverse variance weighted	0.753 (0.467,1.216)	0.246
	Simple mode	1.205 (0.562,2.584)	0.636
	Weighted mode	1.186 (0.59,2.382)	0.637
Cooked vegetable intake	MR Egger	0.022 (0,34.626)	0.337
	Weighted median	2.257 (1.102,4.625)	0.026
	Inverse variance weighted	1.194 (0.619,2.305)	0.597
	Simple mode	2.468 (0.734,8.296)	0.175
	Weighted mode	2.468 (0.787,7.743)	0.152
Salad/raw vegetable intake	MR Egger	0.691 (0.022,21.251)	0.836
	Weighted median	0.983 (0.499,1.938)	0.961
	Inverse variance weighted	0.792 (0.396,1.586)	0.511
	Simple mode	1.37 (0.345,5.432)	0.661
	Weighted mode	1.133 (0.271,4.735)	0.866
Bread intake	MR Egger	1.029 (0.223,4.756)	0.971
	Weighted median	1.228 (0.846,1.783)	0.280
	Inverse variance weighted	1.121 (0.777,1.619)	0.541
	Simple mode	1.487 (0.717,3.087)	0.298
	Weighted mode	1.423 (0.831,2.435)	0.212
Pea intake	MR Egger	0.999 (0.735,1.359)	0.995
	Weighted median	1.133 (0.941,1.364)	0.189
	Inverse variance weighted	1.14 (1.003,1.295)	0.045*
	Simple mode	0.984 (0.651,1.487)	0.940
	Weighted mode	0.975 (0.666,1.428)	0.899
Unsalted peanuts intake	MR Egger	0.515 (0.011,25.289)	0.761
	Weighted median	1.509 (0.351,6.494)	0.580
	Inverse variance weighted	1.3 (0.406,4.168)	0.659
	Simple mode	1.838 (0.28,12.079)	0.561
	Weighted mode	1.764 (0.263,11.815)	0.590
Salted peanuts intake	MR Egger	0.766 (0.443,1.322)	0.347
	Weighted median	1.029 (0.707,1.498)	0.881
	Inverse variance weighted	1.056 (0.776,1.439)	0.727
	Simple mode	0.903 (0.44,1.854)	0.783
	Weighted mode	0.903 (0.429,1.9)	0.790
Milk intake	MR Egger	1.164 (0.597,2.268)	0.659
	Weighted median	1.089 (0.792,1.497)	0.599
	Inverse variance weighted	0.998 (0.734,1.355)	0.988
	Simple mode	1.323 (0.638,2.745)	0.457
	Weighted mode	1.174 (0.595,2.315)	0.647
Yogurt intake	MR Egger	1.321 (0.905,1.927)	0.174
	Weighted median	1.008 (0.796,1.275)	0.950
	Inverse variance weighted	1.046 (0.867,1.263)	0.638
	Simple mode	0.851 (0.569,1.274)	0.448
	Weighted mode	0.864 (0.574,1.3)	0.494
Beef intake	MR Egger	0.848 (0.092,7.851)	0.889
	Weighted median	1.053 (0.561,1.974)	0.873
	Inverse variance weighted	1.024 (0.621,1.687)	0.927
	Simple mode	1.149 (0.418,3.156)	0.796
	Weighted mode	1.107 (0.453,2.707)	0.830
Unsalted nuts intake	MR Egger	1.437 (0.874,2.36)	0.164
	Weighted median	1.121 (0.798,1.574)	0.510
	Inverse variance weighted	1.1 (0.858,1.41)	0.454
	Simple mode	1.188 (0.586,2.408)	0.637
	Weighted mode	1.188 (0.601,2.349)	0.625
Salted nuts intake	MR Egger	1.166 (0.641,2.122)	0.618
	Weighted median	0.964 (0.626,1.484)	0.867
	Inverse variance weighted	0.841 (0.606,1.166)	0.298
	Simple mode	0.984 (0.432,2.243)	0.970
	Weighted mode	0.984 (0.424,2.285)	0.971
Lamb intake	MR Egger	0.62 (0.387,0.994)	0.059
	Weighted median	0.909 (0.652,1.267)	0.573
	Inverse variance weighted	0.836 (0.654,1.067)	0.150
	Simple mode	0.941 (0.498,1.78)	0.853
	Weighted mode	0.929 (0.495,1.745)	0.822
Indian snacks intake	MR Egger	1.249 (0.747,2.088)	0.400
	Weighted median	0.949 (0.658,1.369)	0.779
	Inverse variance weighted	1.067 (0.794,1.436)	0.666
	Simple mode	0.802 (0.342,1.884)	0.615
	Weighted mode	0.827 (0.342,1.995)	0.674
Watercress intake	MR Egger	0.769 (0.451,1.31)	0.344
	Weighted median	0.622 (0.433,0.894)	0.010
	Inverse variance weighted	0.679 (0.51,0.903)	0.008*
	Simple mode	0.502 (0.245,1.031)	0.073
	Weighted mode	0.511 (0.251,1.043)	0.078
Cheese intake	Inverse variance weighted (Multiplicative random effects)	0.737 (0.603,0.901)	0.003*

*p < 0.05. Statistically significant results were obtained using the IVW method. *OR: The 95% Odds Ratio refers to the range of values within which we are 95% confident that the true OR lies, indicating the strength of association between a genetic variant and a health outcome.

To select valid instrumental variables (IVs), we included SNPs that reached the genome-wide significant level (*p* < 5 × 10^−8^) and applied strict cutoff values (R^2^ < 0.01; region size = 5,000 kb) to remove SNPs in linkage disequilibrium. The threshold criteria we selected were based on previous studies (19, 20). For dietary items with less than 5 SNPs meeting the strict threshold (*p* < 5 × 10^−8^), namely peas, salted peanuts, milk, yogurt, unsalted nuts, salted nuts, lamb, Indian snacks, and watercress, we chose to use a relaxed threshold (*p* < 1 × 10^−5^; R^2^ < 0.01; region size = 5,000 kb) to select SNPs. Additionally, SNPs with a minimum allele frequency (MAF) below 0.05 were excluded due to their unstable association with dietary intake. To fulfill the second critical hypothesis, we evaluated the sub-phenotype of the selected SNPs using the PhenoScanner database (*p* < 1 × 10^−5^). We also excluded SNPs associated with body mass index (BMI) and cholesterol, as well as SNPs directly related to OA, to avoid violating the third critical hypothesis that the IVs should not directly relate to the outcome. Furthermore, we ruled out SNPs associated with multiple diets to reduce potential pleiotropy across the SNPs. The OA data came from a previous genome-wide association study (GWAS) (21).

### 2.2 Statistical analysis

This study utilized SNPs to represent the genetic prediction level of dietary intake and investigated their association with the risk of OA. The primary method used was the fixed-effects inverse-variance weighted (IVW) method, which combines Wald utilizes a fixed-effects meta-analysis model to integrate ratio estimates from multiple genetic variants, providing a comprehensive effect estimate of dietary influence on OA (19). By combining Wald estimates for each SNP through a meta-analysis, the IVW method generates an overarching assessment of the diet’s impact on OA. The IVW method can provide unbiased estimations if there is no horizontal pleiotropy imbalance (22).

The weighted median approach, for instance, allowed for the inclusion of half the weight from invalid genetic variants while providing a consistent point estimate. The MR-Egger method, based on the InSIDE hypothesis, which allows for the possibility of pleiotropy under certain conditions where it posits that the strength of the association between the genetic variants (used as IVs) and the exposure is independent of any direct effect these variants might have on the outcome, enables a valid test of the null associational hypothesis and a consistent estimation of associational effects even if all genetic variants are invalid IVs (18, 23).

### 2.3 Sensitivity analysis

In Mendelian Randomization (MR) analysis, conducting heterogeneity and pleiotropy tests is essential to ensure the validity and reliability of causal inferences derived from genetic variants used as instrumental variables. Heterogeneity in MR refers to significant variations in the effects of different genetic variants on the exposure variable, which may signal issues like inappropriate instrumental variables or unaccounted confounding factors. Heterogeneity tests aim to evaluate the consistency of genetic instruments in influencing the outcome, thereby enhancing the accuracy of MR analyses. On the other hand, pleiotropy occurs when a genetic variant impacts multiple traits, potentially influencing the outcome through pathways unrelated to the exposure of interest. Pleiotropy tests strive to detect and adjust for genetic variants that could bias results by affecting the outcome through multiple mechanisms. Together, these tests play a crucial role in minimizing bias, enhancing the precision and reliability of causal estimates in MR studies, and ultimately increasing confidence in the conclusions drawn regarding causal relationships.

Sensitivity analysis was conducted to explore various potential effects in the final model. In each analysis of association between dietary intake and OA, Cochran’s Q statistics were used to measure the heterogeneity between independent variables (24). If heterogeneity was detected (PCochran’s Q < 0.05), then the multiplicative random-effects IVW model was applied to avoid bias toward weaker instrument exposure associations. The MR-Egger intercept test was used to evaluate pleiotropy by comparing the intercept term to zero. A significant difference suggested the presence of horizontal pleiotropy between IVs. Additionally, forest plots, scatter plots, funnel plots, and leave-one-out analysis plots were used to visualize the results with high confidence.

Dietary items with statistically significant IVW results were selected for further study. The *p*-value of IVW results obtained from four items, namely coffee, cheese, peas, and watercress, was found to be less than 0.05. However, the p-value of the heterogeneity test for cheese was lower than 0.05. Therefore, we conducted Mendelian randomization using non-fixed model IVW analysis with IVs of cheese. The non-fixed model IVW considers the variability of instrumental variable effects, allowing for differences in these effects among various genetic mutations. This method typically results in broader confidence intervals that better represent the uncertainty in estimates, thus minimizing the potential for drawing misleading conclusions.

## 3 Results

### 3.1 Statistically significant dietary preferences

**Figure 1:**
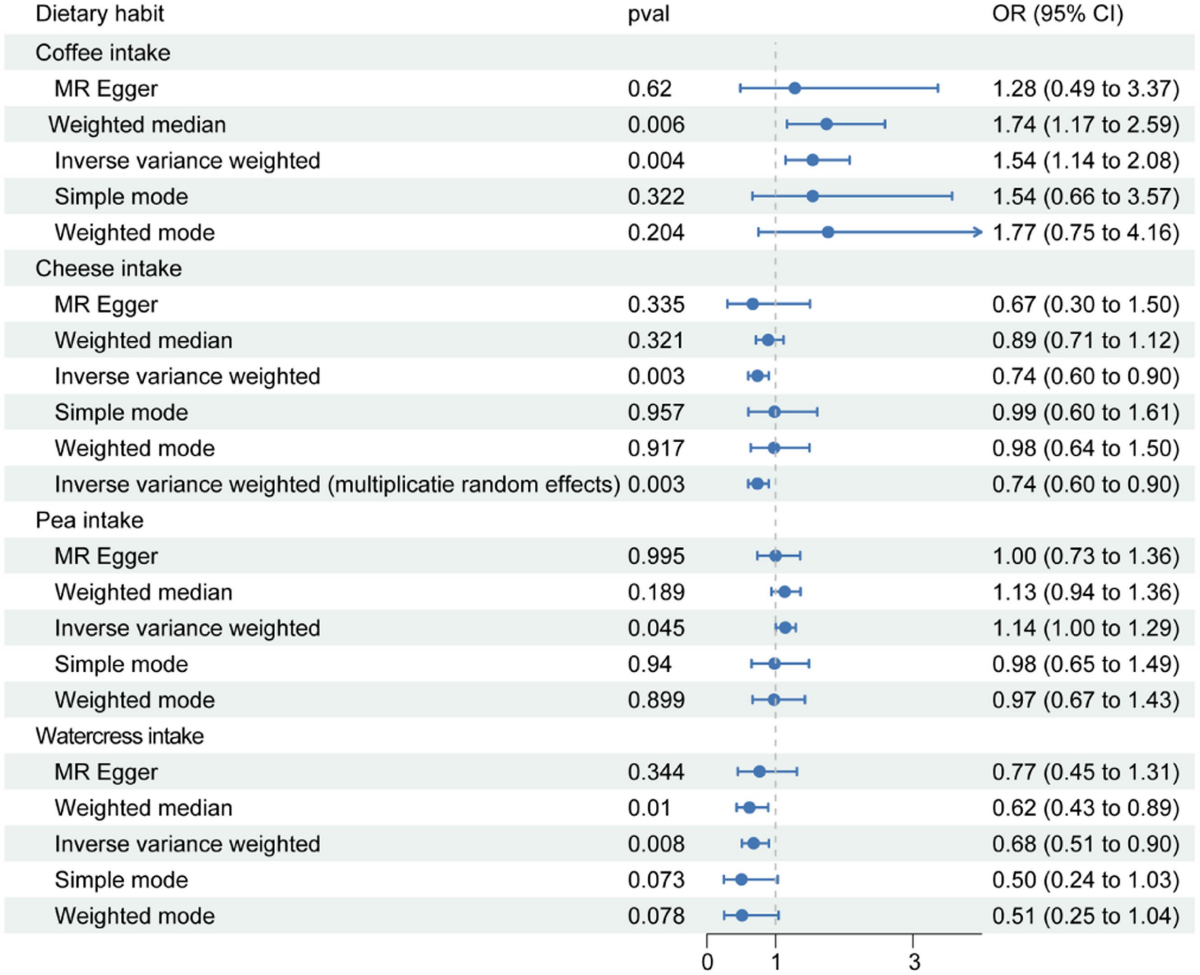
The results of Mendelian randomization analysis of statistically significant dietary preferences were screened, and the results were displayed using forest maps. The term ‘pval’ refers to the *p*-value obtained from the corresponding Mendelian randomization analysis method. The 95% Odds Ratio (OR) refers to the range of values within which we are 95% confident that the true OR lies, indicating the strength of association between a genetic variant and a health outcome.

At the onset of the study, the instrumental variables for 21 dietary exposure factors were individually screened (Supplementary Table S1). MR analysis across 21 different food items in relation to OA indicated statistically significant associations for four dietary preferences, namely coffee, cheese, peas, and watercress. Significant differences were determined based on *p*-values less than 0.05 during data analysis. The inverse-variance weighted (IVW) p-values were found to be 0.004, 0.003, 0.045, and 0.008, respectively (Table 1). The 95% confidence interval for the ratio of coffee was (1.145, 2.079), for cheese was (0.605, 0.901), for peas was (1.003, 1.295), and for watercress was (0.510, 0.903). Coffee and peas were found to have a promoting effect on OA, while cheese and watercress were found to have an inhibiting effect (Figure 1). The results of Mendelian randomization analysis for other food intakes were displayed in the graph. However, the statistical analysis did not reveal a significant relationship between other food groups and osteoarthritis.

### 3.2 Results of sensitivity analysis

The sensitivity analysis results of the 21 dietary items were tabulated (Table 2), showing the heterogeneity and pleiotropy test results of the chosen IVs. Our main focus was on the IVW method and its application to the four specific dietary items that showed significant associations with OA. Furthermore, the heterogeneity test for cheese yielded a *p*-value of 0.002, which was represented using random IVW.

**Table 2:** Sensitivity tests for Mendelian randomization of all dietary preferences.

Exposure	*p*_value*	SNP*	Heterogeneity		Pleiotropy		
			Cochran’s Q statistic	*p*-value	MR-Egger intercept	SE	*p*-value
Coffee intake	5.00E-08	27	30.684	0.240	0.002	0.006	0.694
Tea intake	5.00E-08	33	66.395	0.000*	−0.017	0.008	0.050
Cheese intake	5.00E-08	49	81.692	0.002*	0.002	0.007	0.809
Cereal intake	5.00E-08	25	40.895	0.012*	−0.013	0.014	0.341
Pork intake	5.00E-08	9	13.217	0.105	0.016	0.026	0.557
Fresh fruit intake	5.00E-08	36	44.316	0.134	0.002	0.005	0.714
Dried fruit intake	5.00E-08	25	65.076	0.000*	0.000	0.014	0.975
Cooked vegetable intake	5.00E-08	11	16.675	0.054	0.041	0.038	0.314
Salad/raw vegetable intake	5.00E-08	15	32.302	0.004*	0.001	0.018	0.938
Bread intake	5.00E-08	23	50.900	0.000*	0.001	0.011	0.911
Pea intake	1.00E-05	25	23.649	0.482	0.006	0.007	0.365
Unsalted peanuts intake	5.00E-08	5	1.515	0.824	0.021	0.042	0.659
Salted peanuts intake	1.00E-05	27	34.315	0.127	0.008	0.005	0.177
Milk take	1.00E-05	37	76.704	0.000*	−0.003	0.007	0.613
Yogurt intake	1.00E-05	14	18.520	0.139	−0.010	0.007	0.193
Beef intake	5.00E-08	8	3.839	0.798	0.002	0.014	0.870
Unsalted nuts intake	1.00E-05	28	29.590	0.333	−0.007	0.006	0.235
Salted nuts intake	1.00E-05	38	38.800	0.389	−0.006	0.005	0.210
Lamb intake	1.00E-05	25	13.323	0.961	0.008	0.005	0.161
Indian snacks intake	1.00E-05	56	72.210	0.060	−0.003	0.004	0.465
Watercress intake	1.00E-05	24	28.410	0.201	−0.003	0.006	0.590

*p value: The final threshold, *p*, is determined when selecting the instrumental variable for the exposure factor. *p-value <0.05. The selected instrumental variables were statistically heterogeneous. *SNP: The number of instrument variables selected.

**Figure 2:**
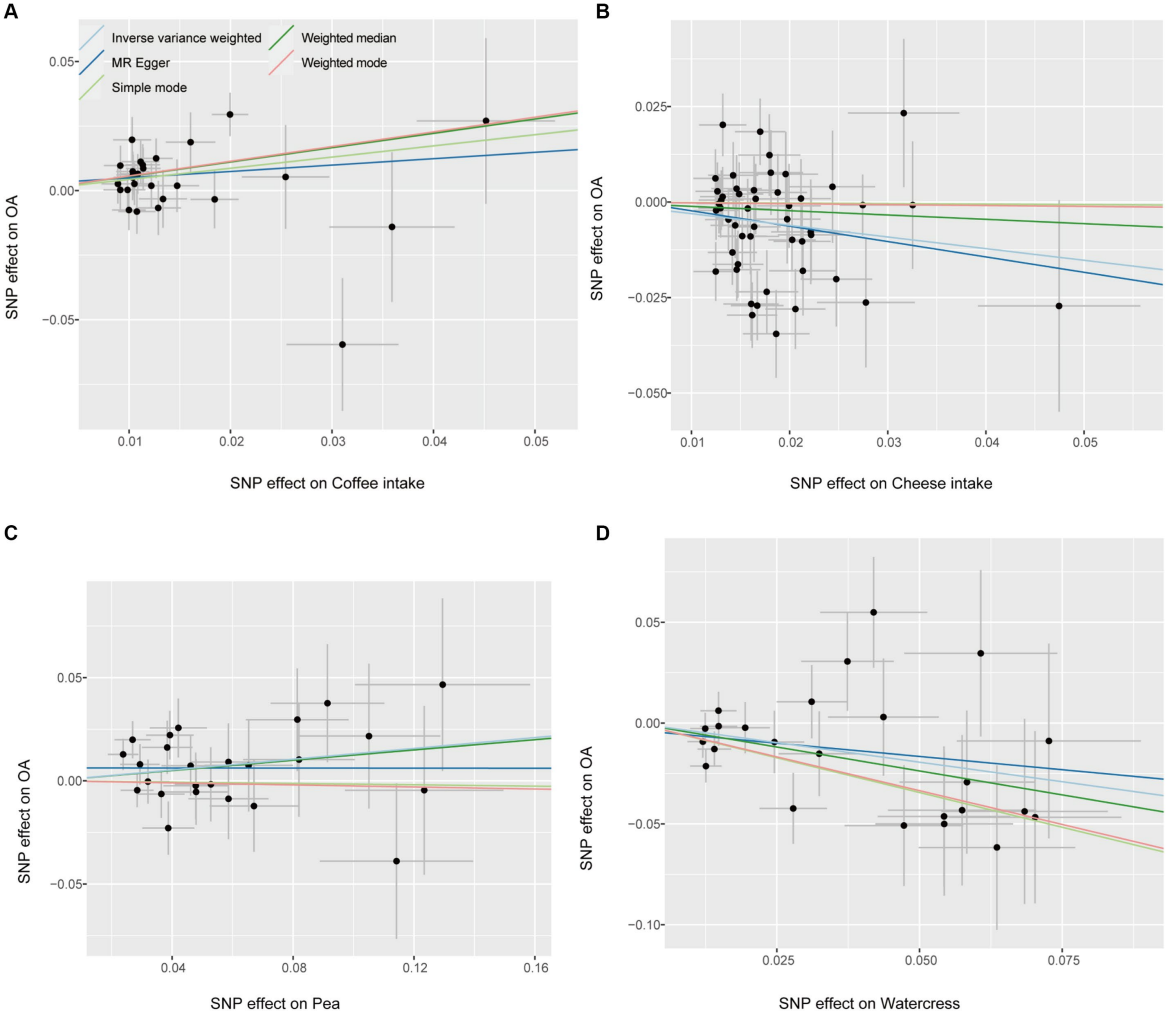
Scatter plot. Each point on the graph represents an IV, and the lines at each point represent the 95% confidence interval, with the horizontal coordinate showing the SNP effect on diet, the vertical coordinate showing the SNP effect on OA, and the colored lines showing the MR Fit. **(A)** Coffee intake **(B)** Cheese intake **(C)** Pea intake **(D)** Watercress intake.

**Figure 3:**
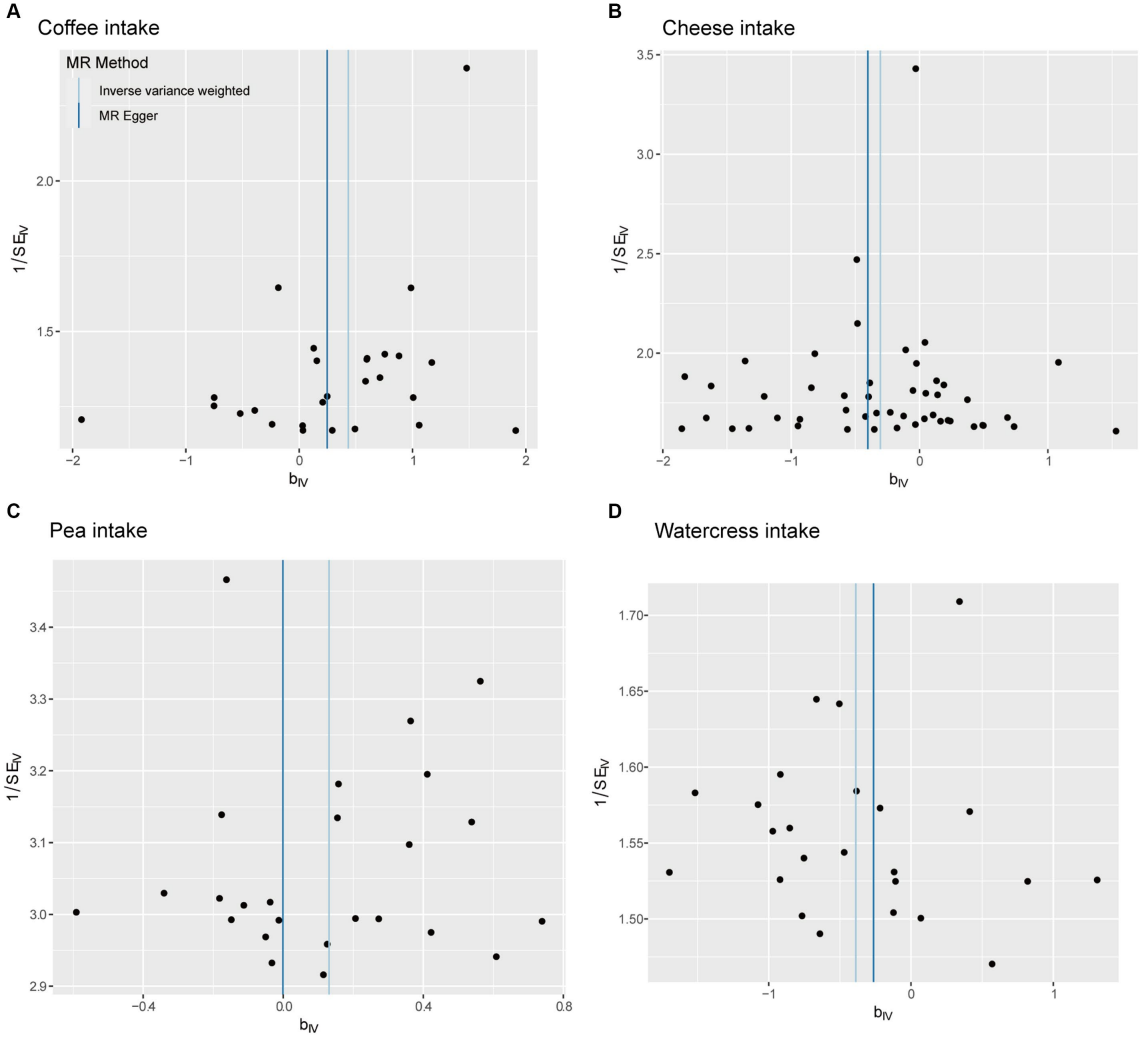
Funnel diagram. The points on the graph reflect the overall distribution of the selected IV. **(A)** Coffee intake, **(B)** cheese intake, **(C)** pea intake, **(D)** watercress intake.

The fitting results of different MR analyses were presented through scatter plots (Figure 2), while funnel plots were used to visually assess the heterogeneity of IVs (Figure 3). Figure 2 displayed the outcomes of an MR analysis investigating the association between exposure factors and outcome factors. The different colors of the lines corresponded to distinct algorithms utilized in the analysis. The results revealed a consistent pattern across the lines for four types of food intake examined by various algorithms, demonstrating a positive relationship between coffee and pea consumption and osteoarthritis. In contrast, cheese and watercress intake were associated with a negative impact on the condition. The funnel plot in Figure 3 illustrated the heterogeneity of the selected SNPs through individual black dots. The symmetrical distribution of these dots around the IVW method in the plot indicated the robustness of the chosen SNPs for our analysis. This symmetry implied that the selected SNPs offered a fair and impartial estimation of the relationship between exposure factors and the outcome, thereby strengthening the credibility of our findings within the realm of Mendelian Randomization studies.

**Figure 4:**
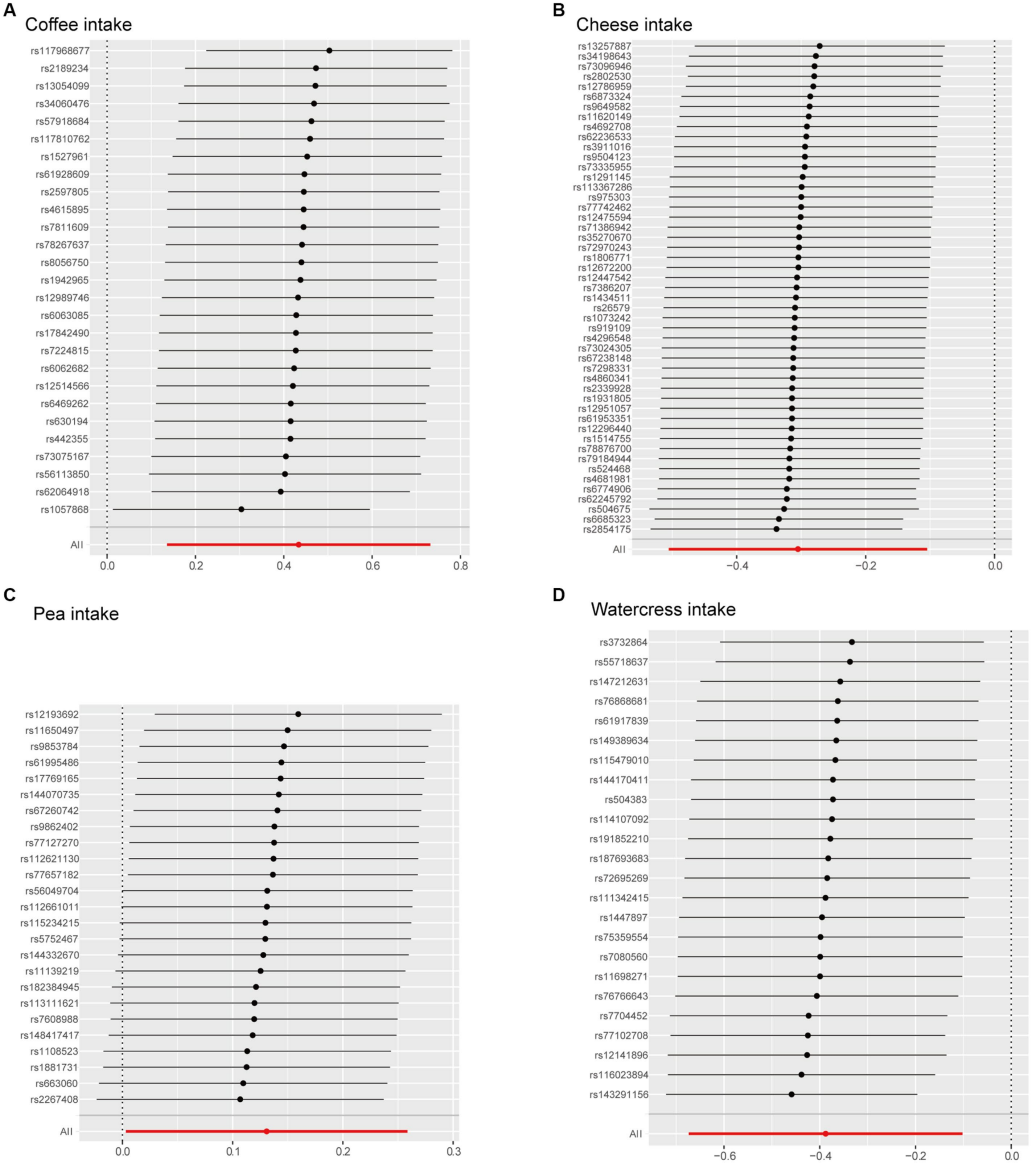
Leave-one-out plot. The Mendelian effect after removing every instrumental variable. **(A)** Coffee intake, **(B)** cheese intake, **(C)** pea intake, **(D)** watercress intake.

To assess the robustness of these results, we performed sensitivity testing using the leave-one-out analysis (Figure 4). The leave-one-out plot revealed that the four IVs of dietary preference identified in IVW exhibited a relatively strong level of consistency. Additionally, the effect of each SNP variable aligned closely with the overall effect. Each point in the leave-one-out plot represents the total effect of other SNPs on osteoarthritis after excluding this particular SNP. Consistency in the impact of excluding each SNP suggested that the effects of the SNPs are relatively consistent. Our findings revealed that the overall effect remains relatively consistent even after excluding each SNP. The results suggested that each SNP related to food intake has an effect on osteoarthritis, with most effects showing consistency, thereby enhancing confidence in our research findings.

## 4 Discussion

Mendelian randomization provides a robust framework to differentiate between correlation and causation, offering more reliable insights compared to conventional observational studies. In this study, we used MR to analyze genetic variants associated with common dietary habits, and inferred the potential causal effects of these dietary items on OA. This research may help inform the development of dietary recommendations and preventive strategies for OA. Interestingly, our findings identified four dietary preferences which may impact OA, namely coffee, peas, watercress, and cheese, where the first two had a promoting effect and the last two an inhibiting effect.

Several studies have pointed to the relationship between coffee and OA (25–27). A cross-sectional study found that consuming more than 7 cups of coffee per day was linked to a higher risk of OA in men, and this risk increased with the amount of coffee consumption (26). A recent review also summarized experimental as well as clinical evidence on the negative effects of caffeine on hyaline cartilage, including its catabolic effects on articular and growth plate cartilage (28). Other studies have suggested an indirect effect of caffeine on the relationship between inflammatory factors and articular cartilage, with caffeine intake being associated with the expression of inflammatory cytokines IL-1 and TNF-α (29).

Pea consumption was found to contribute to OA in our study. Very limited research has explored this association in the literature, with only one study suggesting that alcoholic drinks produced using peas was an independent risk factor for OA (30). It should be noted however that this study set out to investigate the relation of alcohol and specific alcoholic drinks with OA risk, and that high consumption of alcohol itself (>14 standard drinks per week) was significantly associated with incident knee surgery due to OA (30). Further research is needed to determine whether there are specific components in peas that may trigger or enhance molecular pathways leading to OA progression.

Interestingly, our findings suggested an inhibitory effect of watercress on OA, which might be related to several mechanisms. Firstly, watercress contains high levels of beneficial compounds such as isothiocyanates, which have shown strong anti-inflammatory properties (31). This may help reduce inflammation in joint tissues and retard associated pro-inflammatory mechanisms contributing to OA development (32). Secondly, watercress is rich in antioxidants such as vitamin C and beta-carotene, which may counteract the harmful effects of free radicals (31), known to be implicated in the degradation of joint cartilage leading to OA progression (33). Furthermore, the nutrients present in watercress may help protect the integrity of cartilage, maintaining normal cartilage metabolism and inhibiting or slowing catabolic processes (34).

Among dairy products, cheese is naturally nutrient dense and provides high levels of compounds that help maintain bone density and strength, which can be beneficial in OA management especially given the bone weakening associated with OA (35) and the critical role of the subchondral bone in OA (36). Cheese provides a rich source of calcium and is often fortified with vitamin D, both of which are essential for maintaining bone health (37). Some cheeses, especially those made from the milk of grass-fed animals, contain conjugated linoleic acid (CLA), a fatty acid shown to possess anti-inflammatory properties (38). Other cheeses from grass-fed animals can have high levels of omega-3 fatty acids (39), also known for their potent anti-inflammatory properties (40). Both of these compounds may provide benefits in reducing the inflammation involved in the pathogenesis of joint diseases including OA.

In this study, we employed a dual-sample MR approach to investigate potential associations between four specific dietary items and the onset of OA. It is important to acknowledge that certain limitations may exist in our work. Firstly, the observational nature of our study may introduce potential biases, as the establishment of a causative relationship would require randomized controlled trials. Additionally, the genetic instruments used for MR may not fully capture the complexity of dietary influences on OA, considering its multifactorial nature as well as the wide-ranging effects of diet on other factors associated with OA such as exercise, obesity, and co-morbidities including cardiovascular disease (41). Secondly, our findings may have been influenced by unmeasured confounding factors. It only includes a range of genetic markers directly associated with the items investigated in this study. To gain an enhanced and more accurate understanding of the relationship between diet and OA, further studies incorporating a wider array of genetic markers and consideration of environmental factors are necessary, particularly given an emerging understanding of the role of epigenetics in OA progression (42). Ideally, these studies should involve diverse populations to comprehend the intricate interplay among genetics, diet, and lifestyle factors in the context of OA. Due to the utilization of summary GWAS data, the demographic information is restricted, with the population solely reflecting individuals of European descent. Consequently, the generalization of the research findings is constrained. In an investigation into the correlation between 21 dietary intakes and osteoarthritis, our study employed Mendelian randomization to evaluate causality, using *p*-values for statistical significance. Despite its innovative approach, this methodology encounters challenges when dealing with multiple comparisons, especially given the numerous dietary factors involved, which increases the risk of Type I errors. The absence of adjustments for these multiple comparisons represents a notable limitation of our study, underscoring the need for cautious interpretation of our results. Nevertheless, our findings offer valuable insights into the link between diet and osteoarthritis. Future research should consider incorporating correction methods like the Bonferroni or Benjamini-Hochberg procedures to improve result reliability and explore alternative statistical techniques, such as Bayesian approaches, to more precisely evaluate the genuine relationship between diet and osteoarthritis while addressing the issue of multiple comparisons. Moreover, relaxing the threshold for selecting SNPs related to certain dietary items due to a limited number of SNPs meeting the stringent genome-wide significance level may introduce bias and potentially impact the outcomes of our study. Lastly, it is important to note that while the potential mechanisms discussed in this study in relation to the identified food items with significant influence on OA are supported by preliminary research, further studies are necessary to fully understand the extent of the impact of these dietary preferences on OA.

## Data availability statement

The original contributions presented in the study are included in the article/Supplementary material, further inquiries can be directed to the corresponding author.

## Author contributions

LC: Formal analysis, Methodology, Writing – original draft, Writing – review & editing. YS: Formal analysis, Methodology, Writing – review & editing. HL: Methodology, Writing – review & editing. ZY: Methodology, Writing – review & editing. JL: Writing – review & editing. DX: Funding acquisition, Methodology, Supervision, Writing – review & editing.
