# Supplementary material for: The role of dietary preferences in osteoarthritis: a Mendelian randomization study using genome-wide association analysis data from the UK Biobank
Source: Front Nutr. 2024 Apr 29;11:1373850. doi: 10.3389/fnut.2024.1373850 (PMC11089188; doi:10.3389/fnut.2024.1373850)
Supplement: Supplementary file 1 [file Table_1.DOCX]

Supplementary Material

**The Role of Dietary Preferences in Osteoarthritis: A Mendelian Randomization Study Using Genome-wide Association Analysis Data from the UK Biobank**

**Long Chen^1^, Yiqi Su^1^, Hui Li^1^, Zhen Yang^1^, Jiao Jiao Li^2^, Dan Xing^1*^**

^1^Arthritis Clinic and Research Center, Peking University People’s Hospital, Peking University, Beijing 100044, China.

^2^School of Biomedical Engineering, Faculty of Engineering and IT, University of Technology Sydney, Sydney, NSW 2007, Australia.

*** Correspondence:**Dan Xing
xingdan@bjmu.edu.cn

**Contents**

Supplementary Table 1. The summary of information regarding SNPs selected in our study.

Supplementary Table 1. The summary of information regarding SNPs selected in our study.

|  | Exposure \|\|id | Sample size | SNP | Effect_allele | Other_allele | EAF | Beta | Se | p | n | F | R2 |
| --- | --- | --- | --- | --- | --- | --- | --- | --- | --- | --- | --- | --- |
| 1 | Coffee intake \|\| id:ukb-b-5237 | 428,860 | rs10119174 | C | G | 0.571035 | -0.00939786 | 0.00164159 | 1.00E-08 | 428860 | 32.77393185 | 7.64E-05 |
| 2 | Coffee intake \|\| id:ukb-b-5237 |  | rs1057868 | T | C | 0.284986 | 0.0199509 | 0.00178517 | 5.40E-29 | 428860 | 124.9009737 | 0.000291156 |
| 3 | Coffee intake \|\| id:ukb-b-5237 |  | rs117810762 | A | G | 0.017881 | 0.0359086 | 0.00617871 | 6.20E-09 | 428860 | 33.77546389 | 7.88E-05 |
| 4 | Coffee intake \|\| id:ukb-b-5237 |  | rs117968677 | A | G | 0.024207 | -0.0310299 | 0.00551601 | 1.90E-08 | 428860 | 31.6454053 | 7.38E-05 |
| 5 | Coffee intake \|\| id:ukb-b-5237 |  | rs12514566 | A | G | 0.337107 | -0.0113972 | 0.00170562 | 2.40E-11 | 428860 | 44.65106059 | 0.000104105 |
| 6 | Coffee intake \|\| id:ukb-b-5237 |  | rs12989746 | T | G | 0.249928 | 0.01035 | 0.00186429 | 2.80E-08 | 428860 | 30.8214992 | 7.19E-05 |
| 7 | Coffee intake \|\| id:ukb-b-5237 |  | rs13054099 | C | T | 0.261004 | -0.0107777 | 0.00183597 | 4.30E-09 | 428860 | 34.46045604 | 8.03E-05 |
| 8 | Coffee intake \|\| id:ukb-b-5237 |  | rs1527961 | C | T | 0.1349 | -0.0133431 | 0.00236585 | 1.70E-08 | 428860 | 31.80819827 | 7.42E-05 |
| 9 | Coffee intake \|\| id:ukb-b-5237 |  | rs17842490 | G | A | 0.014248 | -0.0451683 | 0.00680848 | 3.30E-11 | 428860 | 44.01160013 | 0.000102615 |
| 10 | Coffee intake \|\| id:ukb-b-5237 |  | rs1942965 | C | T | 0.504585 | -0.00890339 | 0.00161917 | 3.80E-08 | 428860 | 30.23610867 | 7.05E-05 |
| 11 | Coffee intake \|\| id:ukb-b-5237 |  | rs2189234 | G | T | 0.617795 | 0.00998689 | 0.00166052 | 1.80E-09 | 428860 | 36.17198243 | 8.43E-05 |
| 12 | Coffee intake \|\| id:ukb-b-5237 |  | rs2597805 | T | C | 0.682463 | 0.00985502 | 0.00175623 | 2.00E-08 | 428860 | 31.48851914 | 7.34E-05 |
| 13 | Coffee intake \|\| id:ukb-b-5237 |  | rs34060476 | G | A | 0.133855 | 0.0184292 | 0.00237033 | 7.50E-15 | 428860 | 60.44986565 | 0.000140936 |
| 14 | Coffee intake \|\| id:ukb-b-5237 |  | rs442355 | C | G | 0.254435 | -0.0111372 | 0.00185374 | 1.90E-09 | 428860 | 36.09561078 | 8.42E-05 |
| 15 | Coffee intake \|\| id:ukb-b-5237 |  | rs4615895 | A | G | 0.740926 | 0.0122025 | 0.00184972 | 4.20E-11 | 428860 | 43.51967545 | 0.000101468 |
| 16 | Coffee intake \|\| id:ukb-b-5237 |  | rs56113850 | C | T | 0.578109 | 0.0126667 | 0.00163351 | 8.90E-15 | 428860 | 60.12891602 | 0.000140187 |
| 17 | Coffee intake \|\| id:ukb-b-5237 |  | rs57918684 | A | G | 0.154747 | 0.0128864 | 0.00223845 | 8.60E-09 | 428860 | 33.14121451 | 7.73E-05 |
| 18 | Coffee intake \|\| id:ukb-b-5237 |  | rs6062682 | T | C | 0.464546 | 0.0103704 | 0.00163929 | 2.50E-10 | 428860 | 40.02021679 | 9.33E-05 |
| 19 | Coffee intake \|\| id:ukb-b-5237 |  | rs6063085 | C | A | 0.373473 | 0.0104106 | 0.00166919 | 4.50E-10 | 428860 | 38.8991375 | 9.07E-05 |
| 20 | Coffee intake \|\| id:ukb-b-5237 |  | rs61928609 | C | A | 0.835328 | -0.0147305 | 0.00217536 | 1.30E-11 | 428860 | 45.85352204 | 0.000106909 |
| 21 | Coffee intake \|\| id:ukb-b-5237 |  | rs62064918 | T | C | 0.244545 | -0.0103075 | 0.00187872 | 4.10E-08 | 428860 | 30.10111407 | 7.02E-05 |
| 22 | Coffee intake \|\| id:ukb-b-5237 |  | rs630194 | C | T | 0.343374 | -0.0113533 | 0.00169853 | 2.30E-11 | 428860 | 44.67841767 | 0.000104169 |
| 23 | Coffee intake \|\| id:ukb-b-5237 |  | rs6469262 | C | T | 0.564966 | -0.00915347 | 0.00162895 | 1.90E-08 | 428860 | 31.57592028 | 7.36E-05 |
| 24 | Coffee intake \|\| id:ukb-b-5237 |  | rs7224815 | T | A | 0.407832 | -0.0108602 | 0.00164162 | 3.70E-11 | 428860 | 43.76534713 | 0.00010204 |
| 25 | Coffee intake \|\| id:ukb-b-5237 |  | rs73075167 | T | A | 0.12918 | -0.0160639 | 0.00244429 | 5.00E-11 | 428860 | 43.19132446 | 0.000100702 |
| 26 | Coffee intake \|\| id:ukb-b-5237 |  | rs7811609 | T | C | 0.374746 | 0.00913864 | 0.00166468 | 4.00E-08 | 428860 | 30.13711083 | 7.03E-05 |
| 27 | Coffee intake \|\| id:ukb-b-5237 |  | rs78267637 | G | C | 0.038115 | -0.0254259 | 0.00431659 | 3.90E-09 | 428860 | 34.69533367 | 8.09E-05 |
| 28 | Coffee intake \|\| id:ukb-b-5237 |  | rs8056750 | T | C | 0.359129 | 0.0105333 | 0.00173692 | 1.30E-09 | 428860 | 36.77640515 | 8.57E-05 |
| 29 | Tea intake \|\| id:ukb-b-6066 | 447,485 | rs10741694 | C | T | 0.627915 | 0.0150037 | 0.00219355 | 7.90E-12 | 447485 | 46.78446497 | 0.000104539 |
| 30 | Tea intake \|\| id:ukb-b-6066 |  | rs10752269 | A | G | 0.506082 | -0.0128727 | 0.00211975 | 1.30E-09 | 447485 | 36.87822488 | 8.24E-05 |
| 31 | Tea intake \|\| id:ukb-b-6066 |  | rs10764990 | A | G | 0.607155 | -0.0121906 | 0.00216898 | 1.90E-08 | 447485 | 31.58923203 | 7.06E-05 |
| 32 | Tea intake \|\| id:ukb-b-6066 |  | rs11164870 | G | C | 0.604574 | -0.0119604 | 0.00218232 | 4.20E-08 | 447485 | 30.03686088 | 6.71E-05 |
| 33 | Tea intake \|\| id:ukb-b-6066 |  | rs1156588 | G | A | 0.210071 | -0.015454 | 0.00260325 | 2.90E-09 | 447485 | 35.24114916 | 7.87E-05 |
| 34 | Tea intake \|\| id:ukb-b-6066 |  | rs11587444 | G | A | 0.393464 | 0.0140328 | 0.00217078 | 1.00E-10 | 447485 | 41.78852554 | 9.34E-05 |
| 35 | Tea intake \|\| id:ukb-b-6066 |  | rs12591786 | T | C | 0.158804 | -0.0184399 | 0.00294243 | 3.70E-10 | 447485 | 39.27397356 | 8.78E-05 |
| 36 | Tea intake \|\| id:ukb-b-6066 |  | rs13282783 | T | C | 0.285899 | -0.0135837 | 0.00235432 | 7.90E-09 | 447485 | 33.28933955 | 7.44E-05 |
| 37 | Tea intake \|\| id:ukb-b-6066 |  | rs132904 | C | G | 0.778651 | 0.0166007 | 0.00255257 | 7.80E-11 | 447485 | 42.29582311 | 9.45E-05 |
| 38 | Tea intake \|\| id:ukb-b-6066 |  | rs141071726 | A | G | 0.026713 | 0.0407321 | 0.00681204 | 2.20E-09 | 447485 | 35.75355444 | 7.99E-05 |
| 39 | Tea intake \|\| id:ukb-b-6066 |  | rs1453548 | A | T | 0.664929 | -0.0133414 | 0.00224973 | 3.00E-09 | 447485 | 35.16754172 | 7.86E-05 |
| 40 | Tea intake \|\| id:ukb-b-6066 |  | rs149805207 | G | A | 0.008538 | -0.0719337 | 0.0125823 | 1.10E-08 | 447485 | 32.68471685 | 7.30E-05 |
| 41 | Tea intake \|\| id:ukb-b-6066 |  | rs17245213 | A | G | 0.208046 | -0.0146481 | 0.00260905 | 2.00E-08 | 447485 | 31.52084042 | 7.04E-05 |
| 42 | Tea intake \|\| id:ukb-b-6066 |  | rs17576658 | A | G | 0.247081 | -0.0134812 | 0.00245655 | 4.10E-08 | 447485 | 30.11659629 | 6.73E-05 |
| 43 | Tea intake \|\| id:ukb-b-6066 |  | rs17685 | A | G | 0.277512 | 0.0230655 | 0.00236195 | 1.60E-22 | 447485 | 95.36397544 | 0.000213067 |
| 44 | Tea intake \|\| id:ukb-b-6066 |  | rs2117137 | G | A | 0.405148 | 0.0129948 | 0.0021557 | 1.70E-09 | 447485 | 36.33812848 | 8.12E-05 |
| 45 | Tea intake \|\| id:ukb-b-6066 |  | rs2273447 | T | A | 0.203788 | 0.0174715 | 0.00263421 | 3.30E-11 | 447485 | 43.99057077 | 9.83E-05 |
| 46 | Tea intake \|\| id:ukb-b-6066 |  | rs2279844 | A | G | 0.379343 | -0.0119879 | 0.00218318 | 4.00E-08 | 447485 | 30.15137588 | 6.74E-05 |
| 47 | Tea intake \|\| id:ukb-b-6066 |  | rs2351187 | A | G | 0.318935 | 0.0129023 | 0.0022823 | 1.60E-08 | 447485 | 31.95867836 | 7.14E-05 |
| 48 | Tea intake \|\| id:ukb-b-6066 |  | rs2645929 | G | A | 0.813066 | -0.0149842 | 0.0027166 | 3.50E-08 | 447485 | 30.42395917 | 6.80E-05 |
| 49 | Tea intake \|\| id:ukb-b-6066 |  | rs2783129 | G | C | 0.484878 | -0.0117331 | 0.00213314 | 3.80E-08 | 447485 | 30.25428023 | 6.76E-05 |
| 50 | Tea intake \|\| id:ukb-b-6066 |  | rs34619 | A | G | 0.430905 | 0.0117117 | 0.0021375 | 4.30E-08 | 447485 | 30.02117124 | 6.71E-05 |
| 51 | Tea intake \|\| id:ukb-b-6066 |  | rs4817505 | C | T | 0.38998 | 0.015068 | 0.0021746 | 4.20E-12 | 447485 | 48.01229387 | 0.000107283 |
| 52 | Tea intake \|\| id:ukb-b-6066 |  | rs56188862 | C | T | 0.387454 | -0.0157568 | 0.0021747 | 4.30E-13 | 447485 | 52.49734455 | 0.000117303 |
| 53 | Tea intake \|\| id:ukb-b-6066 |  | rs56348300 | G | C | 0.184619 | 0.0158824 | 0.00273191 | 6.10E-09 | 447485 | 33.79866009 | 7.55E-05 |
| 54 | Tea intake \|\| id:ukb-b-6066 |  | rs57462170 | A | G | 0.108773 | 0.0191505 | 0.00340563 | 1.90E-08 | 447485 | 31.62025072 | 7.07E-05 |
| 55 | Tea intake \|\| id:ukb-b-6066 |  | rs57631352 | G | A | 0.296859 | -0.0131035 | 0.00232117 | 1.70E-08 | 447485 | 31.86843439 | 7.12E-05 |
| 56 | Tea intake \|\| id:ukb-b-6066 |  | rs6829 | T | C | 0.596155 | -0.0119163 | 0.00216546 | 3.70E-08 | 447485 | 30.28185843 | 6.77E-05 |
| 57 | Tea intake \|\| id:ukb-b-6066 |  | rs713598 | G | C | 0.402254 | 0.0133969 | 0.00215659 | 5.20E-10 | 447485 | 38.58988258 | 8.62E-05 |
| 58 | Tea intake \|\| id:ukb-b-6066 |  | rs72797284 | G | A | 0.270797 | -0.0171147 | 0.00238353 | 7.00E-13 | 447485 | 51.55815132 | 0.000115205 |
| 59 | Tea intake \|\| id:ukb-b-6066 |  | rs7757102 | G | A | 0.555426 | -0.0118039 | 0.00213302 | 3.10E-08 | 447485 | 30.62394866 | 6.84E-05 |
| 60 | Tea intake \|\| id:ukb-b-6066 |  | rs9624470 | A | G | 0.580054 | 0.0252071 | 0.00215485 | 1.30E-31 | 447485 | 136.8395635 | 0.000305705 |
| 61 | Tea intake \|\| id:ukb-b-6066 |  | rs9648476 | A | G | 0.622954 | 0.0125013 | 0.00218542 | 1.10E-08 | 447485 | 32.72205182 | 7.31E-05 |
| 62 | Tea intake \|\| id:ukb-b-6066 |  | rs977474 | T | C | 0.833746 | 0.0217813 | 0.00285559 | 2.40E-14 | 447485 | 58.18029093 | 0.00013 |
| 63 | Cheese intake \|\| id:ukb-b-1489 | 451,486 | rs1024853 | G | C | 0.437902 | -0.0128634 | 0.00226242 | 1.30E-08 | 451486 | 32.32697644 | 7.16E-05 |
| 64 | Cheese intake \|\| id:ukb-b-1489 |  | rs1073242 | A | G | 0.553825 | 0.015728 | 0.00229156 | 6.70E-12 | 451486 | 47.10690108 | 0.000104327 |
| 65 | Cheese intake \|\| id:ukb-b-1489 |  | rs113367286 | T | C | 0.278467 | 0.0151797 | 0.00249985 | 1.30E-09 | 451486 | 36.87215126 | 8.17E-05 |
| 66 | Cheese intake \|\| id:ukb-b-1489 |  | rs11620149 | C | T | 0.143308 | -0.0176745 | 0.0032085 | 3.60E-08 | 451486 | 30.34521272 | 6.72E-05 |
| 67 | Cheese intake \|\| id:ukb-b-1489 |  | rs12296440 | A | G | 0.169681 | 0.0187942 | 0.00297985 | 2.80E-10 | 451486 | 39.77945989 | 8.81E-05 |
| 68 | Cheese intake \|\| id:ukb-b-1489 |  | rs12447542 | A | G | 0.12558 | 0.0197476 | 0.00340698 | 6.80E-09 | 451486 | 33.59614718 | 7.44E-05 |
| 69 | Cheese intake \|\| id:ukb-b-1489 |  | rs12475594 | G | A | 0.178459 | 0.0160073 | 0.00292518 | 4.40E-08 | 451486 | 29.94545924 | 6.63E-05 |
| 70 | Cheese intake \|\| id:ukb-b-1489 |  | rs12672200 | A | G | 0.325794 | -0.0137634 | 0.00239456 | 9.00E-09 | 451486 | 33.03695536 | 7.32E-05 |
| 71 | Cheese intake \|\| id:ukb-b-1489 |  | rs12786959 | T | A | 0.196267 | -0.0160712 | 0.00281988 | 1.20E-08 | 451486 | 32.48144616 | 7.19E-05 |
| 72 | Cheese intake \|\| id:ukb-b-1489 |  | rs1291145 | C | T | 0.685848 | -0.0202495 | 0.00241026 | 4.40E-17 | 451486 | 70.58311508 | 0.000156311 |
| 73 | Cheese intake \|\| id:ukb-b-1489 |  | rs12951057 | G | C | 0.165627 | -0.0211557 | 0.00304376 | 3.60E-12 | 451486 | 48.30966088 | 0.00010699 |
| 74 | Cheese intake \|\| id:ukb-b-1489 |  | rs13257887 | C | T | 0.358942 | 0.0161808 | 0.00256186 | 2.70E-10 | 451486 | 39.89231082 | 8.84E-05 |
| 75 | Cheese intake \|\| id:ukb-b-1489 |  | rs1434511 | T | C | 0.455286 | 0.0129646 | 0.00225868 | 9.50E-09 | 451486 | 32.94646557 | 7.30E-05 |
| 76 | Cheese intake \|\| id:ukb-b-1489 |  | rs1514755 | G | A | 0.239596 | 0.0163749 | 0.00261651 | 3.90E-10 | 451486 | 39.16629721 | 8.67E-05 |
| 77 | Cheese intake \|\| id:ukb-b-1489 |  | rs1806771 | G | T | 0.087876 | -0.0221467 | 0.004035 | 4.10E-08 | 451486 | 30.12527134 | 6.67E-05 |
| 78 | Cheese intake \|\| id:ukb-b-1489 |  | rs1931805 | C | T | 0.500062 | 0.0126363 | 0.00223591 | 1.60E-08 | 451486 | 31.93972844 | 7.07E-05 |
| 79 | Cheese intake \|\| id:ukb-b-1489 |  | rs2339928 | A | G | 0.704069 | 0.0148578 | 0.00244494 | 1.20E-09 | 451486 | 36.92943 | 8.18E-05 |
| 80 | Cheese intake \|\| id:ukb-b-1489 |  | rs26579 | C | G | 0.586217 | -0.0128007 | 0.00229427 | 2.40E-08 | 451486 | 31.12994736 | 6.89E-05 |
| 81 | Cheese intake \|\| id:ukb-b-1489 |  | rs2802530 | A | G | 0.876502 | 0.0186266 | 0.00339704 | 4.20E-08 | 451486 | 30.06532171 | 6.66E-05 |
| 82 | Cheese intake \|\| id:ukb-b-1489 |  | rs2854175 | A | C | 0.257474 | 0.0169937 | 0.00256857 | 3.70E-11 | 451486 | 43.77166659 | 9.69E-05 |
| 83 | Cheese intake \|\| id:ukb-b-1489 |  | rs34198643 | T | C | 0.224165 | -0.0167006 | 0.00267855 | 4.50E-10 | 451486 | 38.87448361 | 8.61E-05 |
| 84 | Cheese intake \|\| id:ukb-b-1489 |  | rs35270670 | G | A | 0.217962 | 0.0163794 | 0.00270974 | 1.50E-09 | 451486 | 36.53766027 | 8.09E-05 |
| 85 | Cheese intake \|\| id:ukb-b-1489 |  | rs3911016 | G | T | 0.120938 | 0.0213573 | 0.0034399 | 5.30E-10 | 451486 | 38.54792995 | 8.54E-05 |
| 86 | Cheese intake \|\| id:ukb-b-1489 |  | rs4296548 | G | T | 0.609594 | 0.0130245 | 0.00228781 | 1.20E-08 | 451486 | 32.4102374 | 7.18E-05 |
| 87 | Cheese intake \|\| id:ukb-b-1489 |  | rs4681981 | A | C | 0.469086 | -0.0124361 | 0.00224121 | 2.90E-08 | 451486 | 30.78954762 | 6.82E-05 |
| 88 | Cheese intake \|\| id:ukb-b-1489 |  | rs4692708 | C | A | 0.252659 | 0.0147298 | 0.00258904 | 1.30E-08 | 451486 | 32.36802342 | 7.17E-05 |
| 89 | Cheese intake \|\| id:ukb-b-1489 |  | rs4860341 | C | T | 0.928742 | 0.0243652 | 0.00435152 | 2.20E-08 | 451486 | 31.35147611 | 6.94E-05 |
| 90 | Cheese intake \|\| id:ukb-b-1489 |  | rs504675 | T | C | 0.352634 | 0.0274418 | 0.00234182 | 1.00E-31 | 451486 | 137.3150591 | 0.000304049 |
| 91 | Cheese intake \|\| id:ukb-b-1489 |  | rs524468 | G | A | 0.260633 | -0.0142421 | 0.00255161 | 2.40E-08 | 451486 | 31.15440566 | 6.90E-05 |
| 92 | Cheese intake \|\| id:ukb-b-1489 |  | rs61953351 | T | G | 0.250366 | 0.0145932 | 0.00257996 | 1.50E-08 | 451486 | 31.99448498 | 7.09E-05 |
| 93 | Cheese intake \|\| id:ukb-b-1489 |  | rs62236533 | A | G | 0.108791 | 0.0247617 | 0.00364674 | 1.10E-11 | 451486 | 46.105349 | 0.000102109 |
| 94 | Cheese intake \|\| id:ukb-b-1489 |  | rs62245792 | A | T | 0.150049 | -0.0179341 | 0.00316317 | 1.40E-08 | 451486 | 32.14505017 | 7.12E-05 |
| 95 | Cheese intake \|\| id:ukb-b-1489 |  | rs6685323 | T | C | 0.309293 | -0.0131886 | 0.00241592 | 4.80E-08 | 451486 | 29.80110002 | 6.60E-05 |
| 96 | Cheese intake \|\| id:ukb-b-1489 |  | rs67238148 | T | G | 0.217471 | 0.0165432 | 0.00271432 | 1.10E-09 | 451486 | 37.14641847 | 8.23E-05 |
| 97 | Cheese intake \|\| id:ukb-b-1489 |  | rs6774906 | C | A | 0.040653 | 0.0316294 | 0.00567262 | 2.50E-08 | 451486 | 31.08955626 | 6.89E-05 |
| 98 | Cheese intake \|\| id:ukb-b-1489 |  | rs6873324 | C | A | 0.425802 | -0.0124766 | 0.00227073 | 3.90E-08 | 451486 | 30.18988844 | 6.69E-05 |
| 99 | Cheese intake \|\| id:ukb-b-1489 |  | rs71386942 | A | C | 0.268947 | 0.0144554 | 0.00252205 | 9.90E-09 | 451486 | 32.85132137 | 7.28E-05 |
| 100 | Cheese intake \|\| id:ukb-b-1489 |  | rs72970243 | A | G | 0.12044 | 0.0222011 | 0.00340166 | 6.70E-11 | 451486 | 42.59583927 | 9.43E-05 |
| 101 | Cheese intake \|\| id:ukb-b-1489 |  | rs7298331 | C | A | 0.604531 | -0.013175 | 0.00230588 | 1.10E-08 | 451486 | 32.64583963 | 7.23E-05 |
| 102 | Cheese intake \|\| id:ukb-b-1489 |  | rs73024305 | C | G | 0.054789 | 0.0325373 | 0.00492718 | 4.00E-11 | 451486 | 43.60799977 | 9.66E-05 |
| 103 | Cheese intake \|\| id:ukb-b-1489 |  | rs73096946 | C | T | 0.157396 | -0.0205914 | 0.00306664 | 1.90E-11 | 451486 | 45.08646211 | 9.99E-05 |
| 104 | Cheese intake \|\| id:ukb-b-1489 |  | rs73335955 | C | T | 0.05334 | 0.0277726 | 0.00497824 | 2.40E-08 | 451486 | 31.12299753 | 6.89E-05 |
| 105 | Cheese intake \|\| id:ukb-b-1489 |  | rs7386207 | T | C | 0.563517 | -0.0124974 | 0.00226925 | 3.60E-08 | 451486 | 30.33015659 | 6.72E-05 |
| 106 | Cheese intake \|\| id:ukb-b-1489 |  | rs77742462 | G | A | 0.020525 | -0.0474668 | 0.00827804 | 9.80E-09 | 451486 | 32.8794745 | 7.28E-05 |
| 107 | Cheese intake \|\| id:ukb-b-1489 |  | rs78876700 | A | G | 0.137431 | 0.0180965 | 0.00327852 | 3.40E-08 | 451486 | 30.46726708 | 6.75E-05 |
| 108 | Cheese intake \|\| id:ukb-b-1489 |  | rs79184944 | A | T | 0.134346 | 0.0196018 | 0.00328458 | 2.40E-09 | 451486 | 35.61494018 | 7.89E-05 |
| 109 | Cheese intake \|\| id:ukb-b-1489 |  | rs919109 | C | G | 0.138752 | 0.0199382 | 0.00324329 | 7.90E-10 | 451486 | 37.79203953 | 8.37E-05 |
| 110 | Cheese intake \|\| id:ukb-b-1489 |  | rs9504123 | C | A | 0.274743 | 0.0141706 | 0.00250387 | 1.50E-08 | 451486 | 32.02970398 | 7.09E-05 |
| 111 | Cheese intake \|\| id:ukb-b-1489 |  | rs9649582 | T | A | 0.317256 | -0.0146166 | 0.00241153 | 1.40E-09 | 451486 | 36.73731282 | 8.14E-05 |
| 112 | Cheese intake \|\| id:ukb-b-1489 |  | rs975303 | G | A | 0.181313 | 0.0212756 | 0.00290699 | 2.50E-13 | 451486 | 53.56443861 | 0.000118627 |
| 113 | Cereal intake \|\| id:ukb-b-15926 | 441,640 | rs10057775 | C | T | 0.893563 | 0.0200293 | 0.00289378 | 4.50E-12 | 441640 | 47.90717053 | 0.000108464 |
| 114 | Cereal intake \|\| id:ukb-b-15926 |  | rs10837531 | G | C | 0.455002 | 0.0107836 | 0.00179687 | 2.00E-09 | 441640 | 36.01589606 | 8.15E-05 |
| 115 | Cereal intake \|\| id:ukb-b-15926 |  | rs10857964 | C | T | 0.205117 | 0.0140866 | 0.0022053 | 1.70E-10 | 441640 | 40.80158275 | 9.24E-05 |
| 116 | Cereal intake \|\| id:ukb-b-15926 |  | rs11038810 | G | A | 0.644091 | 0.0111343 | 0.00186325 | 2.30E-09 | 441640 | 35.70948423 | 8.09E-05 |
| 117 | Cereal intake \|\| id:ukb-b-15926 |  | rs1104608 | C | G | 0.426239 | 0.0108629 | 0.00181843 | 2.30E-09 | 441640 | 35.68604246 | 8.08E-05 |
| 118 | Cereal intake \|\| id:ukb-b-15926 |  | rs11097340 | T | C | 0.399592 | -0.0115334 | 0.00181501 | 2.10E-10 | 441640 | 40.37910276 | 9.14E-05 |
| 119 | Cereal intake \|\| id:ukb-b-15926 |  | rs112780312 | A | G | 0.274969 | -0.0121483 | 0.0020189 | 1.80E-09 | 441640 | 36.20773852 | 8.20E-05 |
| 120 | Cereal intake \|\| id:ukb-b-15926 |  | rs11670024 | G | A | 0.115508 | 0.0160114 | 0.00279947 | 1.10E-08 | 441640 | 32.71199107 | 7.41E-05 |
| 121 | Cereal intake \|\| id:ukb-b-15926 |  | rs11940694 | G | A | 0.604068 | -0.0126647 | 0.00183426 | 5.00E-12 | 441640 | 47.67251045 | 0.000107933 |
| 122 | Cereal intake \|\| id:ukb-b-15926 |  | rs12354267 | C | T | 0.309145 | 0.0116473 | 0.00193279 | 1.70E-09 | 441640 | 36.31459321 | 8.22E-05 |
| 123 | Cereal intake \|\| id:ukb-b-15926 |  | rs13234131 | G | A | 0.128369 | 0.0170117 | 0.0026602 | 1.60E-10 | 441640 | 40.8946836 | 9.26E-05 |
| 124 | Cereal intake \|\| id:ukb-b-15926 |  | rs184643 | A | G | 0.56672 | -0.0121653 | 0.00180474 | 1.60E-11 | 441640 | 45.43770193 | 0.000102874 |
| 125 | Cereal intake \|\| id:ukb-b-15926 |  | rs1853931 | A | G | 0.531294 | -0.0113368 | 0.00181017 | 3.80E-10 | 441640 | 39.22312969 | 8.88E-05 |
| 126 | Cereal intake \|\| id:ukb-b-15926 |  | rs2450126 | G | A | 0.156746 | -0.0149019 | 0.00245669 | 1.30E-09 | 441640 | 36.79447185 | 8.33E-05 |
| 127 | Cereal intake \|\| id:ukb-b-15926 |  | rs2799849 | T | C | 0.678123 | -0.0123289 | 0.00190573 | 9.80E-11 | 441640 | 41.85293497 | 9.48E-05 |
| 128 | Cereal intake \|\| id:ukb-b-15926 |  | rs2817377 | A | G | 0.537948 | 0.00990084 | 0.00178933 | 3.10E-08 | 441640 | 30.61703973 | 6.93E-05 |
| 129 | Cereal intake \|\| id:ukb-b-15926 |  | rs3115230 | A | C | 0.752001 | -0.0114779 | 0.00207131 | 3.00E-08 | 441640 | 30.70680772 | 6.95E-05 |
| 130 | Cereal intake \|\| id:ukb-b-15926 |  | rs3859193 | A | T | 0.470074 | -0.0103258 | 0.00179925 | 9.50E-09 | 441640 | 32.93551018 | 7.46E-05 |
| 131 | Cereal intake \|\| id:ukb-b-15926 |  | rs4739095 | A | G | 0.765732 | -0.0128656 | 0.00210523 | 9.90E-10 | 441640 | 37.34745298 | 8.46E-05 |
| 132 | Cereal intake \|\| id:ukb-b-15926 |  | rs4797242 | A | C | 0.297207 | 0.0114312 | 0.00194889 | 4.50E-09 | 441640 | 34.4040042 | 7.79E-05 |
| 133 | Cereal intake \|\| id:ukb-b-15926 |  | rs627185 | G | C | 0.544427 | -0.0108269 | 0.00179076 | 1.50E-09 | 441640 | 36.55387994 | 8.28E-05 |
| 134 | Cereal intake \|\| id:ukb-b-15926 |  | rs6510177 | C | T | 0.805589 | -0.0130354 | 0.00228744 | 1.20E-08 | 441640 | 32.47501065 | 7.35E-05 |
| 135 | Cereal intake \|\| id:ukb-b-15926 |  | rs6545770 | T | A | 0.748101 | -0.013728 | 0.00206041 | 2.70E-11 | 441640 | 44.39225873 | 0.000100507 |
| 136 | Cereal intake \|\| id:ukb-b-15926 |  | rs68136852 | A | C | 0.152389 | -0.0141222 | 0.00247848 | 1.20E-08 | 441640 | 32.46638078 | 7.35E-05 |
| 137 | Cereal intake \|\| id:ukb-b-15926 |  | rs6918737 | A | T | 0.234488 | 0.0137325 | 0.00210976 | 7.60E-11 | 441640 | 42.36752626 | 9.59E-05 |
| 138 | Cereal intake \|\| id:ukb-b-15926 |  | rs7040561 | A | T | 0.850635 | -0.0162685 | 0.00252002 | 1.10E-10 | 441640 | 41.67609777 | 9.44E-05 |
| 139 | Cereal intake \|\| id:ukb-b-15926 |  | rs78854891 | C | T | 0.065728 | 0.0221411 | 0.00363312 | 1.10E-09 | 441640 | 37.13974364 | 8.41E-05 |
| 140 | Cereal intake \|\| id:ukb-b-15926 |  | rs79642906 | A | G | 0.083314 | -0.0181581 | 0.00322812 | 1.90E-08 | 441640 | 31.64036363 | 7.16E-05 |
| 141 | Cereal intake \|\| id:ukb-b-15926 |  | rs9846396 | T | C | 0.441556 | 0.0119674 | 0.00180031 | 3.00E-11 | 441640 | 44.18806933 | 0.000100045 |
| 142 | Pork intake \|\| id:ukb-b-5640 | 460,162 | rs10972033 | T | G | 0.45643 | 0.00897557 | 0.00147851 | 1.30E-09 | 460162 | 36.85322886 | 8.01E-05 |
| 143 | Pork intake \|\| id:ukb-b-5640 |  | rs11211124 | C | T | 0.230603 | -0.00995273 | 0.00175408 | 1.40E-08 | 460162 | 32.19479408 | 7.00E-05 |
| 144 | Pork intake \|\| id:ukb-b-5640 |  | rs2387807 | T | C | 0.077893 | -0.0150846 | 0.00274837 | 4.10E-08 | 460162 | 30.12431625 | 6.55E-05 |
| 145 | Pork intake \|\| id:ukb-b-5640 |  | rs254152 | G | C | 0.23498 | -0.0104266 | 0.00174217 | 2.20E-09 | 460162 | 35.81825003 | 7.78E-05 |
| 146 | Pork intake \|\| id:ukb-b-5640 |  | rs34161520 | G | C | 0.160381 | 0.0115919 | 0.0020206 | 9.60E-09 | 460162 | 32.91156654 | 7.15E-05 |
| 147 | Pork intake \|\| id:ukb-b-5640 |  | rs36124222 | C | T | 0.433239 | 0.00840935 | 0.00150083 | 2.10E-08 | 460162 | 31.39509867 | 6.82E-05 |
| 148 | Pork intake \|\| id:ukb-b-5640 |  | rs3964074 | C | T | 0.546932 | -0.00895091 | 0.00148347 | 1.60E-09 | 460162 | 36.40632524 | 7.91E-05 |
| 149 | Pork intake \|\| id:ukb-b-5640 |  | rs4146837 | T | C | 0.455561 | 0.00879226 | 0.0014944 | 4.00E-09 | 460162 | 34.61523839 | 7.52E-05 |
| 150 | Pork intake \|\| id:ukb-b-5640 |  | rs9973426 | G | A | 0.176598 | 0.0110847 | 0.00193678 | 1.00E-08 | 460162 | 32.75573161 | 7.12E-05 |
| 151 | Fresh fruit intake \|\| id:ukb-b-3881 | 446,462 | rs10064431 | C | T | 0.522495 | -0.00757392 | 0.00122365 | 6.00E-10 | 446462 | 38.31130962 | 8.58E-05 |
| 152 | Fresh fruit intake \|\| id:ukb-b-3881 |  | rs10192394 | T | C | 0.528785 | -0.00766096 | 0.0012287 | 4.50E-10 | 446462 | 38.87538231 | 8.71E-05 |
| 153 | Fresh fruit intake \|\| id:ukb-b-3881 |  | rs10249294 | A | G | 0.372973 | 0.0195637 | 0.00126299 | 4.10E-54 | 446462 | 239.9397296 | 0.000537138 |
| 154 | Fresh fruit intake \|\| id:ukb-b-3881 |  | rs10271924 | T | C | 0.492595 | -0.00704676 | 0.00125592 | 2.00E-08 | 446462 | 31.48147035 | 7.05E-05 |
| 155 | Fresh fruit intake \|\| id:ukb-b-3881 |  | rs1051547 | C | T | 0.561537 | -0.00757523 | 0.00124161 | 1.10E-09 | 446462 | 37.22384605 | 8.34E-05 |
| 156 | Fresh fruit intake \|\| id:ukb-b-3881 |  | rs10840126 | G | A | 0.376121 | -0.00771754 | 0.00128576 | 1.90E-09 | 446462 | 36.02781772 | 8.07E-05 |
| 157 | Fresh fruit intake \|\| id:ukb-b-3881 |  | rs11032362 | A | G | 0.090976 | 0.0123929 | 0.00212339 | 5.30E-09 | 446462 | 34.06327143 | 7.63E-05 |
| 158 | Fresh fruit intake \|\| id:ukb-b-3881 |  | rs11248509 | T | A | 0.371237 | 0.00732973 | 0.00126801 | 7.40E-09 | 446462 | 33.41416379 | 7.48E-05 |
| 159 | Fresh fruit intake \|\| id:ukb-b-3881 |  | rs11896330 | A | G | 0.632839 | -0.00844567 | 0.0012744 | 3.40E-11 | 446462 | 43.91942955 | 9.84E-05 |
| 160 | Fresh fruit intake \|\| id:ukb-b-3881 |  | rs12044599 | G | A | 0.21008 | 0.00942073 | 0.0015033 | 3.70E-10 | 446462 | 39.27152797 | 8.80E-05 |
| 161 | Fresh fruit intake \|\| id:ukb-b-3881 |  | rs12536253 | C | G | 0.24901 | -0.00815943 | 0.00141599 | 8.30E-09 | 446462 | 33.20467771 | 7.44E-05 |
| 162 | Fresh fruit intake \|\| id:ukb-b-3881 |  | rs12641371 | T | C | 0.433148 | 0.00791271 | 0.00123354 | 1.40E-10 | 446462 | 41.14755406 | 9.22E-05 |
| 163 | Fresh fruit intake \|\| id:ukb-b-3881 |  | rs12780952 | A | G | 0.286382 | 0.00747457 | 0.00135361 | 3.40E-08 | 446462 | 30.4919584 | 6.83E-05 |
| 164 | Fresh fruit intake \|\| id:ukb-b-3881 |  | rs12885598 | A | G | 0.596671 | 0.00751549 | 0.00124738 | 1.70E-09 | 446462 | 36.30087134 | 8.13E-05 |
| 165 | Fresh fruit intake \|\| id:ukb-b-3881 |  | rs13072255 | C | A | 0.493966 | 0.00898012 | 0.00122355 | 2.10E-13 | 446462 | 53.86675793 | 0.000120638 |
| 166 | Fresh fruit intake \|\| id:ukb-b-3881 |  | rs139042899 | C | A | 0.013469 | 0.035995 | 0.00608106 | 3.20E-09 | 446462 | 35.03690849 | 7.85E-05 |
| 167 | Fresh fruit intake \|\| id:ukb-b-3881 |  | rs17049185 | T | G | 0.267872 | 0.00804043 | 0.00139033 | 7.30E-09 | 446462 | 33.44435024 | 7.49E-05 |
| 168 | Fresh fruit intake \|\| id:ukb-b-3881 |  | rs1866823 | A | G | 0.544469 | 0.00743079 | 0.0012406 | 2.10E-09 | 446462 | 35.87619883 | 8.04E-05 |
| 169 | Fresh fruit intake \|\| id:ukb-b-3881 |  | rs2048522 | T | A | 0.434968 | 0.0095625 | 0.00124771 | 1.80E-14 | 446462 | 58.73751713 | 0.000131545 |
| 170 | Fresh fruit intake \|\| id:ukb-b-3881 |  | rs2093654 | G | A | 0.388133 | 0.00712915 | 0.00125874 | 1.50E-08 | 446462 | 32.07771602 | 7.18E-05 |
| 171 | Fresh fruit intake \|\| id:ukb-b-3881 |  | rs2143081 | A | G | 0.539822 | 0.00832073 | 0.00122891 | 1.30E-11 | 446462 | 45.84402103 | 0.000102673 |
| 172 | Fresh fruit intake \|\| id:ukb-b-3881 |  | rs2302593 | G | C | 0.486625 | 0.00852802 | 0.00122303 | 3.10E-12 | 446462 | 48.62081118 | 0.000108891 |
| 173 | Fresh fruit intake \|\| id:ukb-b-3881 |  | rs2790688 | T | C | 0.154068 | 0.0114466 | 0.00169613 | 1.50E-11 | 446462 | 45.54437451 | 0.000102002 |
| 174 | Fresh fruit intake \|\| id:ukb-b-3881 |  | rs329274 | G | A | 0.485636 | 0.0068183 | 0.00122813 | 2.80E-08 | 446462 | 30.82219272 | 6.90E-05 |
| 175 | Fresh fruit intake \|\| id:ukb-b-3881 |  | rs34162196 | T | C | 0.1008 | -0.0181417 | 0.0020297 | 4.00E-19 | 446462 | 79.88997008 | 0.000178909 |
| 176 | Fresh fruit intake \|\| id:ukb-b-3881 |  | rs4302893 | A | G | 0.334189 | 0.00738857 | 0.00129959 | 1.30E-08 | 446462 | 32.3227321 | 7.24E-05 |
| 177 | Fresh fruit intake \|\| id:ukb-b-3881 |  | rs4953150 | T | C | 0.344088 | -0.00843921 | 0.00129268 | 6.60E-11 | 446462 | 42.62079328 | 9.55E-05 |
| 178 | Fresh fruit intake \|\| id:ukb-b-3881 |  | rs559734 | C | G | 0.711817 | 0.00776806 | 0.00136078 | 1.10E-08 | 446462 | 32.58736969 | 7.30E-05 |
| 179 | Fresh fruit intake \|\| id:ukb-b-3881 |  | rs60452247 | A | G | 0.363079 | 0.00796969 | 0.00126932 | 3.40E-10 | 446462 | 39.42217772 | 8.83E-05 |
| 180 | Fresh fruit intake \|\| id:ukb-b-3881 |  | rs62051554 | A | G | 0.108545 | 0.0116118 | 0.00198143 | 4.60E-09 | 446462 | 34.34326853 | 7.69E-05 |
| 181 | Fresh fruit intake \|\| id:ukb-b-3881 |  | rs6475724 | T | C | 0.727296 | 0.00772163 | 0.00137406 | 1.90E-08 | 446462 | 31.57959681 | 7.07E-05 |
| 182 | Fresh fruit intake \|\| id:ukb-b-3881 |  | rs72974263 | T | C | 0.318282 | 0.00738684 | 0.00131242 | 1.80E-08 | 446462 | 31.67901755 | 7.10E-05 |
| 183 | Fresh fruit intake \|\| id:ukb-b-3881 |  | rs7554485 | C | T | 0.611539 | -0.00801014 | 0.00125414 | 1.70E-10 | 446462 | 40.79323752 | 9.14E-05 |
| 184 | Fresh fruit intake \|\| id:ukb-b-3881 |  | rs78537042 | A | C | 0.086801 | -0.0119259 | 0.00218432 | 4.80E-08 | 446462 | 29.80916441 | 6.68E-05 |
| 185 | Fresh fruit intake \|\| id:ukb-b-3881 |  | rs7982441 | C | T | 0.731883 | -0.00841258 | 0.00137614 | 9.80E-10 | 446462 | 37.37086732 | 8.37E-05 |
| 186 | Fresh fruit intake \|\| id:ukb-b-3881 |  | rs8095324 | G | A | 0.404041 | -0.00694613 | 0.00124903 | 2.70E-08 | 446462 | 30.9271624 | 6.93E-05 |
| 187 | Fresh fruit intake \|\| id:ukb-b-3881 |  | rs862227 | G | A | 0.457858 | -0.0101452 | 0.00122398 | 1.10E-16 | 446462 | 68.70250671 | 0.000153859 |
| 188 | Fresh fruit intake \|\| id:ukb-b-3881 |  | rs9517948 | T | C | 0.451352 | 0.00695315 | 0.00123287 | 1.70E-08 | 446462 | 31.80743346 | 7.12E-05 |
| 189 | Dried fruit intake \|\| id:ukb-b-16576 | 421,764 | rs10129747 | G | A | 0.530254 | 0.00935902 | 0.00168126 | 2.60E-08 | 421764 | 30.98781751 | 7.35E-05 |
| 190 | Dried fruit intake \|\| id:ukb-b-16576 |  | rs10896126 | G | A | 0.303582 | -0.015009 | 0.00181919 | 1.60E-16 | 421764 | 68.0686904 | 0.000161365 |
| 191 | Dried fruit intake \|\| id:ukb-b-16576 |  | rs11152349 | A | G | 0.302856 | 0.00991215 | 0.0018176 | 4.90E-08 | 421764 | 29.73987255 | 7.05E-05 |
| 192 | Dried fruit intake \|\| id:ukb-b-16576 |  | rs11586016 | C | G | 0.371004 | 0.00987818 | 0.00173034 | 1.10E-08 | 421764 | 32.59049647 | 7.73E-05 |
| 193 | Dried fruit intake \|\| id:ukb-b-16576 |  | rs11632215 | C | A | 0.120179 | -0.0141434 | 0.00258382 | 4.40E-08 | 421764 | 29.96285118 | 7.10E-05 |
| 194 | Dried fruit intake \|\| id:ukb-b-16576 |  | rs11811826 | A | T | 0.224231 | 0.0132178 | 0.0020058 | 4.40E-11 | 421764 | 43.42532711 | 0.000102951 |
| 195 | Dried fruit intake \|\| id:ukb-b-16576 |  | rs12137234 | T | C | 0.303772 | 0.0102051 | 0.00183744 | 2.80E-08 | 421764 | 30.84666355 | 7.31E-05 |
| 196 | Dried fruit intake \|\| id:ukb-b-16576 |  | rs1582322 | G | A | 0.604805 | 0.00994346 | 0.00171571 | 6.80E-09 | 421764 | 33.58824381 | 7.96E-05 |
| 197 | Dried fruit intake \|\| id:ukb-b-16576 |  | rs1622515 | G | A | 0.484704 | 0.00991719 | 0.00167077 | 2.90E-09 | 421764 | 35.23253812 | 8.35E-05 |
| 198 | Dried fruit intake \|\| id:ukb-b-16576 |  | rs1648404 | T | C | 0.476112 | 0.00941595 | 0.00167357 | 1.80E-08 | 421764 | 31.65486917 | 7.50E-05 |
| 199 | Dried fruit intake \|\| id:ukb-b-16576 |  | rs17184707 | T | C | 0.212811 | -0.0114381 | 0.00204011 | 2.10E-08 | 421764 | 31.4340695 | 7.45E-05 |
| 200 | Dried fruit intake \|\| id:ukb-b-16576 |  | rs2533273 | A | C | 0.48453 | -0.00987659 | 0.00167714 | 3.90E-09 | 421764 | 34.6797068 | 8.22E-05 |
| 201 | Dried fruit intake \|\| id:ukb-b-16576 |  | rs261809 | G | A | 0.540636 | -0.00962858 | 0.00167902 | 9.80E-09 | 421764 | 32.88612836 | 7.80E-05 |
| 202 | Dried fruit intake \|\| id:ukb-b-16576 |  | rs34162196 | T | C | 0.101001 | -0.0223628 | 0.00277168 | 7.10E-16 | 421764 | 65.09778077 | 0.000154323 |
| 203 | Dried fruit intake \|\| id:ukb-b-16576 |  | rs3764002 | T | C | 0.261416 | 0.0131215 | 0.00190081 | 5.10E-12 | 421764 | 47.6529239 | 0.000112973 |
| 204 | Dried fruit intake \|\| id:ukb-b-16576 |  | rs4140799 | A | G | 0.531856 | 0.0094566 | 0.00167846 | 1.80E-08 | 421764 | 31.7430057 | 7.53E-05 |
| 205 | Dried fruit intake \|\| id:ukb-b-16576 |  | rs57499472 | C | T | 0.404131 | 0.00991231 | 0.00171869 | 8.10E-09 | 421764 | 33.26248192 | 7.89E-05 |
| 206 | Dried fruit intake \|\| id:ukb-b-16576 |  | rs62084586 | C | T | 0.165729 | 0.0133946 | 0.00226174 | 3.20E-09 | 421764 | 35.07309892 | 8.32E-05 |
| 207 | Dried fruit intake \|\| id:ukb-b-16576 |  | rs72720396 | G | A | 0.229157 | 0.0114265 | 0.00198541 | 8.70E-09 | 421764 | 33.1227234 | 7.85E-05 |
| 208 | Dried fruit intake \|\| id:ukb-b-16576 |  | rs746868 | G | C | 0.614662 | -0.0129055 | 0.00171452 | 5.20E-14 | 421764 | 56.65843301 | 0.000134319 |
| 209 | Dried fruit intake \|\| id:ukb-b-16576 |  | rs7808471 | C | T | 0.322136 | -0.0115362 | 0.00178604 | 1.10E-10 | 421764 | 41.71989351 | 9.89E-05 |
| 210 | Dried fruit intake \|\| id:ukb-b-16576 |  | rs7829800 | G | A | 0.671041 | -0.0104463 | 0.00178709 | 5.10E-09 | 421764 | 34.16898975 | 8.10E-05 |
| 211 | Dried fruit intake \|\| id:ukb-b-16576 |  | rs8081370 | T | C | 0.910232 | -0.0166666 | 0.00293795 | 1.40E-08 | 421764 | 32.18142153 | 7.63E-05 |
| 212 | Dried fruit intake \|\| id:ukb-b-16576 |  | rs862227 | G | A | 0.458327 | -0.0091646 | 0.00167238 | 4.30E-08 | 421764 | 30.03012213 | 7.12E-05 |
| 213 | Dried fruit intake \|\| id:ukb-b-16576 |  | rs893856 | A | G | 0.148988 | -0.013361 | 0.00234923 | 1.30E-08 | 421764 | 32.34646694 | 7.67E-05 |
| 214 | Cooked vegetable intake \|\| id:ukb-b-8089 | 448,651 | rs10156602 | G | A | 0.361369 | 0.0110063 | 0.00163687 | 1.80E-11 | 448651 | 45.21206197 | 0.000100764 |
| 215 | Cooked vegetable intake \|\| id:ukb-b-8089 |  | rs10161952 | C | A | 0.31274 | -0.0095798 | 0.00168589 | 1.30E-08 | 448651 | 32.28898664 | 7.20E-05 |
| 216 | Cooked vegetable intake \|\| id:ukb-b-8089 |  | rs11138705 | C | G | 0.757204 | 0.0103715 | 0.00183004 | 1.40E-08 | 448651 | 32.11899728 | 7.16E-05 |
| 217 | Cooked vegetable intake \|\| id:ukb-b-8089 |  | rs1816263 | C | T | 0.280188 | 0.00958122 | 0.00174063 | 3.70E-08 | 448651 | 30.29902797 | 6.75E-05 |
| 218 | Cooked vegetable intake \|\| id:ukb-b-8089 |  | rs2052063 | T | C | 0.515902 | -0.00945755 | 0.0015679 | 1.60E-09 | 448651 | 36.38484857 | 8.11E-05 |
| 219 | Cooked vegetable intake \|\| id:ukb-b-8089 |  | rs2102738 | C | A | 0.172403 | -0.0121578 | 0.00208302 | 5.30E-09 | 448651 | 34.06615436 | 7.59E-05 |
| 220 | Cooked vegetable intake \|\| id:ukb-b-8089 |  | rs2252508 | G | A | 0.480256 | 0.0091021 | 0.00156216 | 5.70E-09 | 448651 | 33.94940589 | 7.57E-05 |
| 221 | Cooked vegetable intake \|\| id:ukb-b-8089 |  | rs2844672 | A | G | 0.624125 | -0.00964215 | 0.00160923 | 2.10E-09 | 448651 | 35.90141171 | 8.00E-05 |
| 222 | Cooked vegetable intake \|\| id:ukb-b-8089 |  | rs28450747 | A | G | 0.23257 | -0.0101604 | 0.0018547 | 4.30E-08 | 448651 | 30.01057162 | 6.69E-05 |
| 223 | Cooked vegetable intake \|\| id:ukb-b-8089 |  | rs34155012 | T | C | 0.227386 | 0.0105584 | 0.00192208 | 3.90E-08 | 448651 | 30.17541854 | 6.73E-05 |
| 224 | Cooked vegetable intake \|\| id:ukb-b-8089 |  | rs838133 | G | A | 0.549575 | 0.0116877 | 0.00161465 | 4.50E-13 | 448651 | 52.39638412 | 0.000116773 |
| 225 | Salad / raw vegetable intake \|\| id:ukb-b-1996 | 435,435 | rs10819082 | A | G | 0.66734 | -0.00916475 | 0.00151342 | 1.40E-09 | 435435 | 36.67096236 | 8.42E-05 |
| 226 | Salad / raw vegetable intake \|\| id:ukb-b-1996 |  | rs12203592 | T | C | 0.219338 | -0.0102862 | 0.00169411 | 1.30E-09 | 435435 | 36.86605923 | 8.47E-05 |
| 227 | Salad / raw vegetable intake \|\| id:ukb-b-1996 |  | rs12908495 | A | C | 0.242533 | -0.00935 | 0.00166573 | 2.00E-08 | 435435 | 31.50750448 | 7.24E-05 |
| 228 | Salad / raw vegetable intake \|\| id:ukb-b-1996 |  | rs13102393 | G | C | 0.499077 | 0.00797933 | 0.00143029 | 2.40E-08 | 435435 | 31.12322902 | 7.15E-05 |
| 229 | Salad / raw vegetable intake \|\| id:ukb-b-1996 |  | rs17460017 | T | A | 0.190124 | 0.0111744 | 0.00181338 | 7.20E-10 | 435435 | 37.97263927 | 8.72E-05 |
| 230 | Salad / raw vegetable intake \|\| id:ukb-b-1996 |  | rs1890012 | G | T | 0.19473 | -0.0104258 | 0.00180794 | 8.10E-09 | 435435 | 33.25452525 | 7.64E-05 |
| 231 | Salad / raw vegetable intake \|\| id:ukb-b-1996 |  | rs2194027 | A | T | 0.484664 | -0.00860795 | 0.00143565 | 2.00E-09 | 435435 | 35.95028361 | 8.26E-05 |
| 232 | Salad / raw vegetable intake \|\| id:ukb-b-1996 |  | rs3095337 | C | G | 0.203909 | -0.0126218 | 0.00176637 | 9.00E-13 | 435435 | 51.05981399 | 0.000117248 |
| 233 | Salad / raw vegetable intake \|\| id:ukb-b-1996 |  | rs3129962 | A | G | 0.129168 | -0.0133033 | 0.00212261 | 3.70E-10 | 435435 | 39.28062216 | 9.02E-05 |
| 234 | Salad / raw vegetable intake \|\| id:ukb-b-1996 |  | rs3129962 | A | G | 0.129168 | -0.0133033 | 0.00212261 | 3.70E-10 | 435435 | 39.28062216 | 9.02E-05 |
| 235 | Salad / raw vegetable intake \|\| id:ukb-b-1996 |  | rs34186148 | C | G | 0.370054 | -0.00805142 | 0.0014749 | 4.80E-08 | 435435 | 29.80024351 | 6.84E-05 |
| 236 | Salad / raw vegetable intake \|\| id:ukb-b-1996 |  | rs4083969 | G | C | 0.057217 | 0.017133 | 0.00311517 | 3.80E-08 | 435435 | 30.24846399 | 6.95E-05 |
| 237 | Salad / raw vegetable intake \|\| id:ukb-b-1996 |  | rs4291983 | A | C | 0.517576 | -0.00839985 | 0.00142481 | 3.70E-09 | 435435 | 34.75594909 | 7.98E-05 |
| 238 | Salad / raw vegetable intake \|\| id:ukb-b-1996 |  | rs57221424 | G | C | 0.321733 | 0.00894101 | 0.00153327 | 5.50E-09 | 435435 | 34.00446016 | 7.81E-05 |
| 239 | Salad / raw vegetable intake \|\| id:ukb-b-1996 |  | rs62461186 | C | A | 0.179827 | -0.0113438 | 0.00185732 | 1.00E-09 | 435435 | 37.30299064 | 8.57E-05 |
| 240 | Salad / raw vegetable intake \|\| id:ukb-b-1996 |  | rs75248709 | T | C | 0.045947 | -0.0197339 | 0.00352774 | 2.20E-08 | 435435 | 31.29195574 | 7.19E-05 |
| 241 | Salad / raw vegetable intake \|\| id:ukb-b-1996 |  | rs7821179 | C | G | 0.846577 | -0.0108184 | 0.00197644 | 4.40E-08 | 435435 | 29.96117219 | 6.88E-05 |
| 242 | Salad / raw vegetable intake \|\| id:ukb-b-1996 |  | rs8130508 | A | G | 0.289686 | 0.0087404 | 0.00157797 | 3.00E-08 | 435435 | 30.68069068 | 7.05E-05 |
| 243 | Salad / raw vegetable intake \|\| id:ukb-b-1996 |  | rs9427220 | T | A | 0.554661 | -0.00800801 | 0.00144212 | 2.80E-08 | 435435 | 30.83517474 | 7.08E-05 |
| 244 | Bread intake \|\| id:ukb-b-11348 | 452,236 | rs10761661 | T | C | 0.45322 | -0.0114852 | 0.00200486 | 1.00E-08 | 452236 | 32.81776663 | 7.26E-05 |
| 245 | Bread intake \|\| id:ukb-b-11348 |  | rs11183201 | C | T | 0.507856 | -0.016725 | 0.00199574 | 5.30E-17 | 452236 | 70.23026857 | 0.000155272 |
| 246 | Bread intake \|\| id:ukb-b-11348 |  | rs11628639 | C | T | 0.243333 | -0.013495 | 0.00232226 | 6.20E-09 | 452236 | 33.76945832 | 7.47E-05 |
| 247 | Bread intake \|\| id:ukb-b-11348 |  | rs13016665 | A | C | 0.423235 | 0.0147544 | 0.00202829 | 3.50E-13 | 452236 | 52.9155126 | 0.000116995 |
| 248 | Bread intake \|\| id:ukb-b-11348 |  | rs13023099 | A | C | 0.571784 | -0.0114708 | 0.00202287 | 1.40E-08 | 452236 | 32.15521872 | 7.11E-05 |
| 249 | Bread intake \|\| id:ukb-b-11348 |  | rs1492988 | G | C | 0.599095 | 0.0115447 | 0.00203549 | 1.40E-08 | 452236 | 32.16824424 | 7.11E-05 |
| 250 | Bread intake \|\| id:ukb-b-11348 |  | rs17083079 | A | G | 0.047423 | 0.0301138 | 0.00467938 | 1.20E-10 | 452236 | 41.41469758 | 9.16E-05 |
| 251 | Bread intake \|\| id:ukb-b-11348 |  | rs1940033 | T | C | 0.592727 | -0.011079 | 0.00202843 | 4.70E-08 | 452236 | 29.831911 | 6.60E-05 |
| 252 | Bread intake \|\| id:ukb-b-11348 |  | rs2068650 | C | A | 0.472074 | -0.013938 | 0.00199929 | 3.10E-12 | 452236 | 48.60146191 | 0.000107458 |
| 253 | Bread intake \|\| id:ukb-b-11348 |  | rs2517678 | T | C | 0.368395 | 0.013233 | 0.00208598 | 2.20E-10 | 452236 | 40.24355561 | 8.90E-05 |
| 254 | Bread intake \|\| id:ukb-b-11348 |  | rs28406095 | A | G | 0.461729 | -0.010949 | 0.00200012 | 4.40E-08 | 452236 | 29.96655416 | 6.63E-05 |
| 255 | Bread intake \|\| id:ukb-b-11348 |  | rs4984685 | A | G | 0.201006 | 0.0135849 | 0.00248083 | 4.40E-08 | 452236 | 29.98602382 | 6.63E-05 |
| 256 | Bread intake \|\| id:ukb-b-11348 |  | rs55745436 | T | C | 0.237279 | 0.0134245 | 0.00234263 | 1.00E-08 | 452236 | 32.83891785 | 7.26E-05 |
| 257 | Bread intake \|\| id:ukb-b-11348 |  | rs596878 | C | A | 0.449595 | -0.011739 | 0.00201069 | 5.30E-09 | 452236 | 34.08568053 | 7.54E-05 |
| 258 | Bread intake \|\| id:ukb-b-11348 |  | rs62091167 | C | A | 0.215931 | -0.0138402 | 0.0024253 | 1.20E-08 | 452236 | 32.56520365 | 7.20E-05 |
| 259 | Bread intake \|\| id:ukb-b-11348 |  | rs6580721 | G | A | 0.188665 | 0.017205 | 0.00254355 | 1.30E-11 | 452236 | 45.7539713 | 0.000101163 |
| 260 | Bread intake \|\| id:ukb-b-11348 |  | rs73802707 | T | C | 0.153694 | -0.0159296 | 0.00276165 | 8.00E-09 | 452236 | 33.2715066 | 7.36E-05 |
| 261 | Bread intake \|\| id:ukb-b-11348 |  | rs75287965 | A | G | 0.062933 | -0.0247475 | 0.00409745 | 1.50E-09 | 452236 | 36.47836298 | 8.07E-05 |
| 262 | Bread intake \|\| id:ukb-b-11348 |  | rs7802468 | T | C | 0.371501 | -0.0233527 | 0.00205629 | 6.90E-30 | 452236 | 128.9749813 | 0.000285114 |
| 263 | Bread intake \|\| id:ukb-b-11348 |  | rs79436018 | C | T | 0.116276 | -0.0176147 | 0.00311645 | 1.60E-08 | 452236 | 31.94700686 | 7.06E-05 |
| 264 | Bread intake \|\| id:ukb-b-11348 |  | rs9323989 | C | T | 0.379169 | -0.0116143 | 0.00205547 | 1.60E-08 | 452236 | 31.92741773 | 7.06E-05 |
| 265 | Bread intake \|\| id:ukb-b-11348 |  | rs9529024 | T | A | 0.370396 | -0.0129169 | 0.00206792 | 4.20E-10 | 452236 | 39.01657363 | 8.63E-05 |
| 266 | Bread intake \|\| id:ukb-b-11348 |  | rs9662365 | T | C | 0.499481 | 0.0121661 | 0.00198921 | 9.60E-10 | 452236 | 37.40601951 | 8.27E-05 |
| 267 | Bread intake \|\| id:ukb-b-11348 |  | rs9832088 | A | T | 0.521617 | 0.0147289 | 0.00198902 | 1.30E-13 | 452236 | 54.83556556 | 0.00012124 |
| 268 | Bread intake \|\| id:ukb-b-11348 |  | rs9881332 | G | C | 0.581804 | 0.0113702 | 0.00202188 | 1.90E-08 | 452236 | 31.62463015 | 6.99E-05 |
| 269 | Pea intake \|\| id:ukb-b-15945 | 64,949 | rs1108523 | G | A | 0.473722 | -0.023778 | 0.00504393 | 2.40E-06 | 64949 | 22.22350442 | 0.000342062 |
| 270 | Pea intake \|\| id:ukb-b-15945 |  | rs11139219 | T | C | 0.188707 | 0.0293405 | 0.00653564 | 7.10E-06 | 64949 | 20.15388503 | 0.000310217 |
| 271 | Pea intake \|\| id:ukb-b-15945 |  | rs112621130 | A | G | 0.049311 | 0.0527851 | 0.0119319 | 9.70E-06 | 64949 | 19.57057057 | 0.000301241 |
| 272 | Pea intake \|\| id:ukb-b-15945 |  | rs112661011 | C | T | 0.021744 | 0.0819511 | 0.0184661 | 9.10E-06 | 64949 | 19.69514618 | 0.000303158 |
| 273 | Pea intake \|\| id:ukb-b-15945 |  | rs113111621 | A | G | 0.030902 | 0.0814057 | 0.0169867 | 1.60E-06 | 64949 | 22.96632977 | 0.000353491 |
| 274 | Pea intake \|\| id:ukb-b-15945 |  | rs115234215 | A | C | 0.047552 | -0.0586125 | 0.0124831 | 2.70E-06 | 64949 | 22.04629388 | 0.000339335 |
| 275 | Pea intake \|\| id:ukb-b-15945 |  | rs11650497 | C | T | 0.010576 | 0.114222 | 0.0253628 | 6.70E-06 | 64949 | 20.28173605 | 0.000312184 |
| 276 | Pea intake \|\| id:ukb-b-15945 |  | rs12193692 | G | T | 0.904183 | 0.0387413 | 0.00862922 | 7.10E-06 | 64949 | 20.15604396 | 0.00031025 |
| 277 | Pea intake \|\| id:ukb-b-15945 |  | rs144070735 | G | T | 0.039779 | -0.0586562 | 0.0132539 | 9.60E-06 | 64949 | 19.58576183 | 0.000301474 |
| 278 | Pea intake \|\| id:ukb-b-15945 |  | rs144332670 | G | A | 0.012631 | 0.105101 | 0.0236161 | 8.60E-06 | 64949 | 19.8060257 | 0.000304864 |
| 279 | Pea intake \|\| id:ukb-b-15945 |  | rs148417417 | C | T | 0.018873 | -0.091381 | 0.0188305 | 1.20E-06 | 64949 | 23.54984803 | 0.00036247 |
| 280 | Pea intake \|\| id:ukb-b-15945 |  | rs17769165 | T | C | 0.030786 | 0.0670954 | 0.0146899 | 4.90E-06 | 64949 | 20.8616096 | 0.000321107 |
| 281 | Pea intake \|\| id:ukb-b-15945 |  | rs182384945 | C | T | 0.008166 | -0.129461 | 0.0289587 | 7.80E-06 | 64949 | 19.98571965 | 0.000307629 |
| 282 | Pea intake \|\| id:ukb-b-15945 |  | rs1881731 | G | A | 0.922801 | -0.0420593 | 0.00945527 | 8.70E-06 | 64949 | 19.78682948 | 0.000304568 |
| 283 | Pea intake \|\| id:ukb-b-15945 |  | rs2267408 | G | C | 0.72642 | -0.0269137 | 0.00597544 | 6.70E-06 | 64949 | 20.28649575 | 0.000312257 |
| 284 | Pea intake \|\| id:ukb-b-15945 |  | rs56049704 | T | C | 0.031261 | -0.0653182 | 0.0146266 | 8.00E-06 | 64949 | 19.94259396 | 0.000306965 |
| 285 | Pea intake \|\| id:ukb-b-15945 |  | rs5752467 | A | G | 0.075697 | -0.0461334 | 0.00963706 | 1.70E-06 | 64949 | 22.91615759 | 0.00035272 |
| 286 | Pea intake \|\| id:ukb-b-15945 |  | rs61995486 | C | T | 0.123364 | -0.0364115 | 0.00772781 | 2.50E-06 | 64949 | 22.20057737 | 0.000341709 |
| 287 | Pea intake \|\| id:ukb-b-15945 |  | rs663060 | A | T | 0.116321 | 0.0392321 | 0.00786367 | 6.10E-07 | 64949 | 24.89043869 | 0.000383095 |
| 288 | Pea intake \|\| id:ukb-b-15945 |  | rs67260742 | G | C | 0.061793 | 0.0479039 | 0.0105223 | 5.30E-06 | 64949 | 20.72623341 | 0.000319024 |
| 289 | Pea intake \|\| id:ukb-b-15945 |  | rs6794936 | T | A | 0.529032 | 0.0235476 | 0.00505954 | 3.30E-06 | 64949 | 21.6606374 | 0.000333401 |
| 290 | Pea intake \|\| id:ukb-b-15945 |  | rs7608988 | C | T | 0.100517 | -0.0383765 | 0.00839718 | 4.90E-06 | 64949 | 20.88641016 | 0.000321488 |
| 291 | Pea intake \|\| id:ukb-b-15945 |  | rs77127270 | A | G | 0.009808 | 0.123401 | 0.0262183 | 2.50E-06 | 64949 | 22.1527834 | 0.000340974 |
| 292 | Pea intake \|\| id:ukb-b-15945 |  | rs77657182 | T | G | 0.149339 | 0.0320132 | 0.00716337 | 7.90E-06 | 64949 | 19.97208511 | 0.000307419 |
| 293 | Pea intake \|\| id:ukb-b-15945 |  | rs9853784 | G | T | 0.308303 | -0.0284208 | 0.00546303 | 2.00E-07 | 64949 | 27.06483763 | 0.000416548 |
| 294 | Pea intake \|\| id:ukb-b-15945 |  | rs9862402 | A | G | 0.058792 | 0.0477934 | 0.0106977 | 7.90E-06 | 64949 | 19.95974667 | 0.000307229 |
| 295 | Unsalted peanuts intake \|\| id:ukb-b-15555 | 64,949 | rs112608685 | G | A | 0.043812 | 0.0139597 | 0.00249963 | 2.30E-08 | 64949 | 31.1889471 | 0.000479991 |
| 296 | Unsalted peanuts intake \|\| id:ukb-b-15555 |  | rs116588489 | T | C | 0.01428 | 0.0242147 | 0.00418489 | 7.20E-09 | 64949 | 33.48035788 | 0.000515237 |
| 297 | Unsalted peanuts intake \|\| id:ukb-b-15555 |  | rs117116541 | C | T | 0.013977 | 0.0239199 | 0.00428859 | 2.40E-08 | 64949 | 31.10925868 | 0.000478765 |
| 298 | Unsalted peanuts intake \|\| id:ukb-b-15555 |  | rs140323162 | T | C | 0.008978 | 0.0340413 | 0.00561229 | 1.30E-09 | 64949 | 36.79019299 | 0.000566144 |
| 299 | Unsalted peanuts intake \|\| id:ukb-b-15555 |  | rs958108 | A | C | 0.01611 | 0.0223962 | 0.00398133 | 1.90E-08 | 64949 | 31.6440689 | 0.000486992 |
| 300 | Salted peanuts intake \|\| id:ukb-b-1099 | 64,949 | rs1006217 | C | T | 0.161788 | -0.0136992 | 0.00307477 | 8.40E-06 | 64949 | 19.85021167 | 0.000305544 |
| 301 | Salted peanuts intake \|\| id:ukb-b-1099 |  | rs11137480 | C | G | 0.370397 | 0.0115883 | 0.00235633 | 8.70E-07 | 64949 | 24.18617742 | 0.00037226 |
| 302 | Salted peanuts intake \|\| id:ukb-b-1099 |  | rs115185270 | A | G | 0.012662 | 0.0493528 | 0.0110202 | 7.50E-06 | 64949 | 20.0560148 | 0.000308711 |
| 303 | Salted peanuts intake \|\| id:ukb-b-1099 |  | rs117611360 | A | C | 0.02455 | -0.0336973 | 0.00731256 | 4.10E-06 | 64949 | 21.23495396 | 0.000326851 |
| 304 | Salted peanuts intake \|\| id:ukb-b-1099 |  | rs117879712 | G | A | 0.009731 | 0.0516199 | 0.0115378 | 7.70E-06 | 64949 | 20.01650755 | 0.000308103 |
| 305 | Salted peanuts intake \|\| id:ukb-b-1099 |  | rs118079864 | C | T | 0.008992 | 0.054181 | 0.012042 | 6.80E-06 | 64949 | 20.24402137 | 0.000311604 |
| 306 | Salted peanuts intake \|\| id:ukb-b-1099 |  | rs1209910 | G | C | 0.408054 | 0.0112297 | 0.0023288 | 1.40E-06 | 64949 | 23.25262157 | 0.000357896 |
| 307 | Salted peanuts intake \|\| id:ukb-b-1099 |  | rs1346077 | G | A | 0.100629 | 0.0172754 | 0.00387888 | 8.40E-06 | 64949 | 19.83551751 | 0.000305318 |
| 308 | Salted peanuts intake \|\| id:ukb-b-1099 |  | rs138462855 | G | A | 0.009124 | 0.0642174 | 0.0121175 | 1.20E-07 | 64949 | 28.08532019 | 0.000432248 |
| 309 | Salted peanuts intake \|\| id:ukb-b-1099 |  | rs142627973 | C | T | 0.006979 | 0.0711526 | 0.0146224 | 1.10E-06 | 64949 | 23.67795706 | 0.000364441 |
| 310 | Salted peanuts intake \|\| id:ukb-b-1099 |  | rs142985236 | G | A | 0.019014 | 0.0391228 | 0.00878176 | 8.40E-06 | 64949 | 19.84708506 | 0.000305496 |
| 311 | Salted peanuts intake \|\| id:ukb-b-1099 |  | rs144283007 | A | G | 0.018246 | 0.0422933 | 0.00928492 | 5.20E-06 | 64949 | 20.74850518 | 0.000319366 |
| 312 | Salted peanuts intake \|\| id:ukb-b-1099 |  | rs144387937 | T | C | 0.00728 | 0.0793857 | 0.0140647 | 1.70E-08 | 64949 | 31.85837434 | 0.000490288 |
| 313 | Salted peanuts intake \|\| id:ukb-b-1099 |  | rs145233287 | A | G | 0.010602 | 0.0523782 | 0.0116154 | 6.50E-06 | 64949 | 20.33446726 | 0.000312995 |
| 314 | Salted peanuts intake \|\| id:ukb-b-1099 |  | rs148206324 | A | T | 0.011301 | 0.0504085 | 0.0112683 | 7.70E-06 | 64949 | 20.01201165 | 0.000308033 |
| 315 | Salted peanuts intake \|\| id:ukb-b-1099 |  | rs148780562 | A | G | 0.010246 | 0.0520683 | 0.0115657 | 6.70E-06 | 64949 | 20.26762923 | 0.000311967 |
| 316 | Salted peanuts intake \|\| id:ukb-b-1099 |  | rs181745116 | A | G | 0.013829 | 0.0486855 | 0.0101636 | 1.70E-06 | 64949 | 22.94584946 | 0.000353176 |
| 317 | Salted peanuts intake \|\| id:ukb-b-1099 |  | rs2289964 | T | C | 0.121633 | 0.0159126 | 0.00350138 | 5.50E-06 | 64949 | 20.6539822 | 0.000317912 |
| 318 | Salted peanuts intake \|\| id:ukb-b-1099 |  | rs28639739 | A | G | 0.529249 | -0.0118931 | 0.00230437 | 2.50E-07 | 64949 | 26.63702485 | 0.000409967 |
| 319 | Salted peanuts intake \|\| id:ukb-b-1099 |  | rs28868570 | G | A | 0.018557 | 0.0405353 | 0.0084445 | 1.60E-06 | 64949 | 23.04193266 | 0.000354655 |
| 320 | Salted peanuts intake \|\| id:ukb-b-1099 |  | rs435571 | G | A | 0.056553 | 0.0237383 | 0.0050504 | 2.60E-06 | 64949 | 22.09264304 | 0.000340049 |
| 321 | Salted peanuts intake \|\| id:ukb-b-1099 |  | rs6975809 | G | C | 0.16143 | -0.0143997 | 0.00313403 | 4.30E-06 | 64949 | 21.11060062 | 0.000324938 |
| 322 | Salted peanuts intake \|\| id:ukb-b-1099 |  | rs7190023 | G | C | 0.361454 | 0.0114629 | 0.00236495 | 1.30E-06 | 64949 | 23.49335715 | 0.0003616 |
| 323 | Salted peanuts intake \|\| id:ukb-b-1099 |  | rs72732705 | G | A | 0.01154 | 0.0480715 | 0.0105923 | 5.70E-06 | 64949 | 20.59656528 | 0.000317028 |
| 324 | Salted peanuts intake \|\| id:ukb-b-1099 |  | rs7612138 | C | T | 0.581771 | -0.0102876 | 0.00229498 | 7.40E-06 | 64949 | 20.0941819 | 0.000309298 |
| 325 | Salted peanuts intake \|\| id:ukb-b-1099 |  | rs922863 | A | G | 0.151696 | 0.0145136 | 0.00315047 | 4.10E-06 | 64949 | 21.22264953 | 0.000326662 |
| 326 | Salted peanuts intake \|\| id:ukb-b-1099 |  | rs9421416 | A | G | 0.046814 | 0.0262452 | 0.00573955 | 4.80E-06 | 64949 | 20.9095211 | 0.000321844 |
| 327 | Milk intake \|\| id:ukb-b-2966 | 64,943 | rs113396977 | G | T | 0.041104 | 0.0290502 | 0.00646881 | 7.10E-06 | 64943 | 20.16737913 | 0.000310453 |
| 328 | Milk intake \|\| id:ukb-b-2966 |  | rs114264277 | A | G | 0.015344 | 0.0457144 | 0.0101606 | 6.80E-06 | 64943 | 20.24264874 | 0.000311611 |
| 329 | Milk intake \|\| id:ukb-b-2966 |  | rs11519229 | C | T | 0.035726 | 0.0302379 | 0.00675713 | 7.60E-06 | 64943 | 20.02528493 | 0.000308266 |
| 330 | Milk intake \|\| id:ukb-b-2966 |  | rs115526621 | T | C | 0.026708 | 0.0359507 | 0.00785697 | 4.70E-06 | 64943 | 20.93652077 | 0.000322289 |
| 331 | Milk intake \|\| id:ukb-b-2966 |  | rs116885893 | C | T | 0.022096 | 0.0382168 | 0.00850838 | 7.10E-06 | 64943 | 20.17506223 | 0.000310571 |
| 332 | Milk intake \|\| id:ukb-b-2966 |  | rs117941098 | C | T | 0.056382 | 0.0249399 | 0.00549825 | 5.70E-06 | 64943 | 20.5750287 | 0.000316726 |
| 333 | Milk intake \|\| id:ukb-b-2966 |  | rs117951732 | G | T | 0.030505 | 0.0348826 | 0.00749339 | 3.20E-06 | 64943 | 21.67010537 | 0.000333578 |
| 334 | Milk intake \|\| id:ukb-b-2966 |  | rs12947049 | A | C | 0.599701 | -0.0118263 | 0.00255596 | 3.70E-06 | 64943 | 21.40866948 | 0.000329555 |
| 335 | Milk intake \|\| id:ukb-b-2966 |  | rs13009696 | T | C | 0.119877 | 0.0174729 | 0.00383851 | 5.30E-06 | 64943 | 20.72070988 | 0.000318968 |
| 336 | Milk intake \|\| id:ukb-b-2966 |  | rs13072001 | C | A | 0.014389 | 0.0516626 | 0.0109414 | 2.30E-06 | 64943 | 22.29496151 | 0.000343193 |
| 337 | Milk intake \|\| id:ukb-b-2966 |  | rs138964765 | C | G | 0.009287 | 0.0588423 | 0.0131974 | 8.20E-06 | 64943 | 19.87936556 | 0.000306021 |
| 338 | Milk intake \|\| id:ukb-b-2966 |  | rs140837911 | C | T | 0.019955 | 0.041487 | 0.00921461 | 6.70E-06 | 64943 | 20.27076529 | 0.000312044 |
| 339 | Milk intake \|\| id:ukb-b-2966 |  | rs143934486 | G | A | 0.014525 | 0.049561 | 0.0105993 | 2.90E-06 | 64943 | 21.86380535 | 0.000336559 |
| 340 | Milk intake \|\| id:ukb-b-2966 |  | rs145140220 | G | A | 0.023614 | 0.0388396 | 0.00846466 | 4.50E-06 | 64943 | 21.0537984 | 0.000324094 |
| 341 | Milk intake \|\| id:ukb-b-2966 |  | rs1549862 | C | A | 0.317875 | -0.0125871 | 0.00268764 | 2.80E-06 | 64943 | 21.93356232 | 0.000337632 |
| 342 | Milk intake \|\| id:ukb-b-2966 |  | rs181158113 | C | T | 0.013793 | 0.0544322 | 0.0120767 | 6.60E-06 | 64943 | 20.31492481 | 0.000312723 |
| 343 | Milk intake \|\| id:ukb-b-2966 |  | rs191749866 | C | T | 0.01121 | 0.0643908 | 0.012243 | 1.40E-07 | 64943 | 27.66125898 | 0.000425763 |
| 344 | Milk intake \|\| id:ukb-b-2966 |  | rs191821864 | G | A | 0.021078 | 0.0430578 | 0.00919504 | 2.80E-06 | 64943 | 21.92786893 | 0.000337544 |
| 345 | Milk intake \|\| id:ukb-b-2966 |  | rs2807888 | A | G | 0.84204 | -0.016638 | 0.00343062 | 1.20E-06 | 64943 | 23.52106649 | 0.00036206 |
| 346 | Milk intake \|\| id:ukb-b-2966 |  | rs34980790 | C | T | 0.362062 | 0.0128655 | 0.0026175 | 8.90E-07 | 64943 | 24.15905584 | 0.000371877 |
| 347 | Milk intake \|\| id:ukb-b-2966 |  | rs35780054 | G | A | 0.268283 | 0.0126101 | 0.00282009 | 7.80E-06 | 64943 | 19.99452651 | 0.000307793 |
| 348 | Milk intake \|\| id:ukb-b-2966 |  | rs3744761 | T | C | 0.044365 | 0.030039 | 0.00609529 | 8.30E-07 | 64943 | 24.28746543 | 0.000373853 |
| 349 | Milk intake \|\| id:ukb-b-2966 |  | rs5022342 | G | A | 0.400444 | 0.0115678 | 0.00255429 | 5.90E-06 | 64943 | 20.50978635 | 0.000315722 |
| 350 | Milk intake \|\| id:ukb-b-2966 |  | rs55806675 | A | G | 0.28432 | -0.0125897 | 0.00279716 | 6.80E-06 | 64943 | 20.25798031 | 0.000311847 |
| 351 | Milk intake \|\| id:ukb-b-2966 |  | rs56674454 | C | T | 0.218843 | 0.0150401 | 0.00303001 | 6.90E-07 | 64943 | 24.63844666 | 0.000379254 |
| 352 | Milk intake \|\| id:ukb-b-2966 |  | rs6025776 | A | C | 0.273824 | 0.0129441 | 0.00281142 | 4.10E-06 | 64943 | 21.19787168 | 0.000326311 |
| 353 | Milk intake \|\| id:ukb-b-2966 |  | rs62435191 | A | G | 0.138374 | 0.0178798 | 0.0036392 | 9.00E-07 | 64943 | 24.13867711 | 0.000371564 |
| 354 | Milk intake \|\| id:ukb-b-2966 |  | rs6489968 | A | G | 0.888279 | -0.0182786 | 0.00395456 | 3.80E-06 | 64943 | 21.36434191 | 0.000328873 |
| 355 | Milk intake \|\| id:ukb-b-2966 |  | rs6858396 | A | C | 0.853525 | -0.0158455 | 0.00355913 | 8.50E-06 | 64943 | 19.82093754 | 0.000305121 |
| 356 | Milk intake \|\| id:ukb-b-2966 |  | rs71413244 | A | T | 0.021434 | 0.0433072 | 0.00882584 | 9.30E-07 | 64943 | 24.07731813 | 0.000370619 |
| 357 | Milk intake \|\| id:ukb-b-2966 |  | rs7166313 | C | T | 0.053753 | 0.0245886 | 0.00553604 | 8.90E-06 | 64943 | 19.72736887 | 0.000303681 |
| 358 | Milk intake \|\| id:ukb-b-2966 |  | rs73164589 | A | G | 0.03199 | 0.0329761 | 0.00717061 | 4.20E-06 | 64943 | 21.14883155 | 0.000325556 |
| 359 | Milk intake \|\| id:ukb-b-2966 |  | rs76396593 | T | C | 0.057334 | 0.024319 | 0.00544708 | 8.00E-06 | 64943 | 19.93259846 | 0.00030684 |
| 360 | Milk intake \|\| id:ukb-b-2966 |  | rs77953747 | T | C | 0.006964 | 0.0706124 | 0.0155793 | 5.80E-06 | 64943 | 20.543104 | 0.000316235 |
| 361 | Milk intake \|\| id:ukb-b-2966 |  | rs781770 | T | C | 0.191487 | -0.0154353 | 0.00316901 | 1.10E-06 | 64943 | 23.72372769 | 0.000365179 |
| 362 | Milk intake \|\| id:ukb-b-2966 |  | rs9342975 | A | C | 0.693387 | -0.0131524 | 0.00279613 | 2.60E-06 | 64943 | 22.12561233 | 0.000340587 |
| 363 | Milk intake \|\| id:ukb-b-2966 |  | rs9400375 | G | A | 0.329046 | -0.0127513 | 0.00273945 | 3.20E-06 | 64943 | 21.66617121 | 0.000333517 |
| 364 | Yogurt intake \|\| id:ukb-b-7753 | 64,949 | rs10505667 | T | C | 0.436 | -0.0243369 | 0.00536649 | 5.80E-06 | 64949 | 20.56600165 | 0.000316558 |
| 365 | Yogurt intake \|\| id:ukb-b-7753 |  | rs113524166 | T | G | 0.01086 | 0.12696 | 0.0279812 | 5.70E-06 | 64949 | 20.58738357 | 0.000316887 |
| 366 | Yogurt intake \|\| id:ukb-b-7753 |  | rs113580100 | G | A | 0.04376 | 0.0631919 | 0.0134028 | 2.40E-06 | 64949 | 22.22960538 | 0.000342156 |
| 367 | Yogurt intake \|\| id:ukb-b-7753 |  | rs11521361 | A | T | 0.206901 | 0.0326692 | 0.00657514 | 6.70E-07 | 64949 | 24.68692459 | 0.000379964 |
| 368 | Yogurt intake \|\| id:ukb-b-7753 |  | rs11678849 | A | T | 0.309133 | 0.0277087 | 0.00597292 | 3.50E-06 | 64949 | 21.52082447 | 0.00033125 |
| 369 | Yogurt intake \|\| id:ukb-b-7753 |  | rs144143483 | C | A | 0.013671 | 0.115312 | 0.0251596 | 4.60E-06 | 64949 | 21.00591216 | 0.000323327 |
| 370 | Yogurt intake \|\| id:ukb-b-7753 |  | rs149870452 | C | T | 0.018477 | -0.0970167 | 0.0200442 | 1.30E-06 | 64949 | 23.42693871 | 0.000360578 |
| 371 | Yogurt intake \|\| id:ukb-b-7753 |  | rs150992808 | A | G | 0.037124 | 0.0668096 | 0.014184 | 2.50E-06 | 64949 | 22.18606586 | 0.000341486 |
| 372 | Yogurt intake \|\| id:ukb-b-7753 |  | rs2344658 | A | C | 0.006762 | 0.205586 | 0.0399966 | 2.70E-07 | 64949 | 26.42049342 | 0.000406635 |
| 373 | Yogurt intake \|\| id:ukb-b-7753 |  | rs2344658 | A | C | 0.006762 | 0.205586 | 0.0399966 | 2.70E-07 | 64949 | 26.42049342 | 0.000406635 |
| 374 | Yogurt intake \|\| id:ukb-b-7753 |  | rs2819017 | T | C | 0.098369 | 0.0417101 | 0.00891559 | 2.90E-06 | 64949 | 21.8868008 | 0.000336881 |
| 375 | Yogurt intake \|\| id:ukb-b-7753 |  | rs28614087 | A | C | 0.447425 | 0.0244925 | 0.00539422 | 5.60E-06 | 64949 | 20.61621447 | 0.000317331 |
| 376 | Yogurt intake \|\| id:ukb-b-7753 |  | rs3741434 | C | T | 0.140872 | -0.0355099 | 0.00760024 | 3.00E-06 | 64949 | 21.82952498 | 0.000336 |
| 377 | Yogurt intake \|\| id:ukb-b-7753 |  | rs392542 | A | G | 0.657947 | 0.0274013 | 0.00559887 | 9.90E-07 | 64949 | 23.95198812 | 0.000368657 |
| 378 | Yogurt intake \|\| id:ukb-b-7753 |  | rs7157038 | C | T | 0.321506 | 0.0260636 | 0.00569264 | 4.70E-06 | 64949 | 20.96241736 | 0.000322658 |
| 379 | Beef intake \|\| id:ukb-b-2862 | 461,053 | rs1105388 | T | C | 0.300147 | -0.0113754 | 0.00187563 | 1.30E-09 | 461053 | 36.78231107 | 7.98E-05 |
| 380 | Beef intake \|\| id:ukb-b-2862 |  | rs11165829 | G | C | 0.35997 | -0.0102001 | 0.00177865 | 9.80E-09 | 461053 | 32.88727317 | 7.13E-05 |
| 381 | Beef intake \|\| id:ukb-b-2862 |  | rs132901 | T | C | 0.787662 | 0.0139249 | 0.00209282 | 2.90E-11 | 461053 | 44.27111051 | 9.60E-05 |
| 382 | Beef intake \|\| id:ukb-b-2862 |  | rs1470610 | C | G | 0.196185 | -0.0122159 | 0.00215779 | 1.50E-08 | 461053 | 32.05033591 | 6.95E-05 |
| 383 | Beef intake \|\| id:ukb-b-2862 |  | rs4676964 | T | C | 0.510605 | 0.0133533 | 0.00172438 | 9.60E-15 | 461053 | 59.96685299 | 0.000130049 |
| 384 | Beef intake \|\| id:ukb-b-2862 |  | rs7791463 | A | G | 0.534781 | 0.00954846 | 0.00171063 | 2.40E-08 | 461053 | 31.15691847 | 6.76E-05 |
| 385 | Beef intake \|\| id:ukb-b-2862 |  | rs784251 | T | C | 0.477649 | -0.0103387 | 0.00171651 | 1.70E-09 | 461053 | 36.27765374 | 7.87E-05 |
| 386 | Beef intake \|\| id:ukb-b-2862 |  | rs79809011 | A | G | 0.029465 | -0.0280593 | 0.00508482 | 3.40E-08 | 461053 | 30.45106577 | 6.60E-05 |
| 387 | Unsalted nuts intake \|\| id:ukb-b-12217 | 64,949 | rs11241419 | C | G | 0.068244 | 0.025021 | 0.00506994 | 8.00E-07 | 64949 | 24.35587219 | 0.000374871 |
| 388 | Unsalted nuts intake \|\| id:ukb-b-12217 |  | rs112734217 | T | A | 0.038438 | 0.032244 | 0.00675554 | 1.80E-06 | 64949 | 22.7812571 | 0.000350644 |
| 389 | Unsalted nuts intake \|\| id:ukb-b-12217 |  | rs116123358 | T | C | 0.058903 | -0.0245752 | 0.00553738 | 9.10E-06 | 64949 | 19.696337 | 0.000303176 |
| 390 | Unsalted nuts intake \|\| id:ukb-b-12217 |  | rs117713405 | A | T | 0.006297 | 0.083463 | 0.0170891 | 1.00E-06 | 64949 | 23.85336203 | 0.000367139 |
| 391 | Unsalted nuts intake \|\| id:ukb-b-12217 |  | rs117987918 | A | G | 0.014655 | 0.0514303 | 0.0110851 | 3.50E-06 | 64949 | 21.52577937 | 0.000331326 |
| 392 | Unsalted nuts intake \|\| id:ukb-b-12217 |  | rs138364334 | A | T | 0.007288 | 0.0726857 | 0.0159191 | 5.00E-06 | 64949 | 20.84783364 | 0.000320895 |
| 393 | Unsalted nuts intake \|\| id:ukb-b-12217 |  | rs139312083 | C | T | 0.014654 | 0.0525489 | 0.010873 | 1.30E-06 | 64949 | 23.3576143 | 0.000359512 |
| 394 | Unsalted nuts intake \|\| id:ukb-b-12217 |  | rs140760808 | G | C | 0.007015 | 0.0752483 | 0.0162129 | 3.50E-06 | 64949 | 21.5413034 | 0.000331565 |
| 395 | Unsalted nuts intake \|\| id:ukb-b-12217 |  | rs145713169 | A | G | 0.030457 | 0.0354878 | 0.00756972 | 2.80E-06 | 64949 | 21.97852397 | 0.000338293 |
| 396 | Unsalted nuts intake \|\| id:ukb-b-12217 |  | rs146575647 | C | T | 0.007249 | 0.0788781 | 0.0161265 | 1.00E-06 | 64949 | 23.92393644 | 0.000368225 |
| 397 | Unsalted nuts intake \|\| id:ukb-b-12217 |  | rs181883277 | T | G | 0.015807 | 0.0481654 | 0.0106597 | 6.20E-06 | 64949 | 20.41645698 | 0.000314257 |
| 398 | Unsalted nuts intake \|\| id:ukb-b-12217 |  | rs185589623 | G | A | 0.013421 | 0.0543001 | 0.0120769 | 6.90E-06 | 64949 | 20.21577142 | 0.000311169 |
| 399 | Unsalted nuts intake \|\| id:ukb-b-12217 |  | rs189375034 | A | G | 0.018912 | 0.0426137 | 0.00955062 | 8.10E-06 | 64949 | 19.90835411 | 0.000306438 |
| 400 | Unsalted nuts intake \|\| id:ukb-b-12217 |  | rs2178664 | C | T | 0.037859 | 0.0315804 | 0.00690238 | 4.80E-06 | 64949 | 20.93329159 | 0.00032221 |
| 401 | Unsalted nuts intake \|\| id:ukb-b-12217 |  | rs2664108 | G | A | 0.690161 | -0.0128132 | 0.00279645 | 4.60E-06 | 64949 | 20.99428521 | 0.000323148 |
| 402 | Unsalted nuts intake \|\| id:ukb-b-12217 |  | rs2740789 | T | G | 0.419953 | 0.0117433 | 0.00260281 | 6.40E-06 | 64949 | 20.35613761 | 0.000313329 |
| 403 | Unsalted nuts intake \|\| id:ukb-b-12217 |  | rs2955753 | A | G | 0.456366 | -0.0119348 | 0.00261506 | 5.00E-06 | 64949 | 20.82893073 | 0.000320604 |
| 404 | Unsalted nuts intake \|\| id:ukb-b-12217 |  | rs4734720 | C | T | 0.084401 | 0.0213049 | 0.00459356 | 3.50E-06 | 64949 | 21.51098083 | 0.000331099 |
| 405 | Unsalted nuts intake \|\| id:ukb-b-12217 |  | rs4904335 | G | A | 0.092895 | -0.0205676 | 0.00442179 | 3.30E-06 | 64949 | 21.63570288 | 0.000333018 |
| 406 | Unsalted nuts intake \|\| id:ukb-b-12217 |  | rs56009601 | C | T | 0.11594 | 0.0180648 | 0.00401489 | 6.80E-06 | 64949 | 20.24505745 | 0.000311619 |
| 407 | Unsalted nuts intake \|\| id:ukb-b-12217 |  | rs62030847 | T | C | 0.057422 | 0.0252204 | 0.00549367 | 4.40E-06 | 64949 | 21.07554452 | 0.000324398 |
| 408 | Unsalted nuts intake \|\| id:ukb-b-12217 |  | rs62560375 | T | C | 0.07722 | 0.0213355 | 0.00480886 | 9.10E-06 | 64949 | 19.68436379 | 0.000302992 |
| 409 | Unsalted nuts intake \|\| id:ukb-b-12217 |  | rs67790793 | A | G | 0.137194 | 0.0167533 | 0.00375265 | 8.00E-06 | 64949 | 19.93079441 | 0.000306784 |
| 410 | Unsalted nuts intake \|\| id:ukb-b-12217 |  | rs72824620 | T | C | 0.138131 | 0.0169108 | 0.00371934 | 5.40E-06 | 64949 | 20.67266871 | 0.000318199 |
| 411 | Unsalted nuts intake \|\| id:ukb-b-12217 |  | rs73913809 | A | G | 0.064174 | 0.0240023 | 0.00524212 | 4.70E-06 | 64949 | 20.96485208 | 0.000322695 |
| 412 | Unsalted nuts intake \|\| id:ukb-b-12217 |  | rs743815 | C | T | 0.603699 | -0.0119441 | 0.00267246 | 7.80E-06 | 64949 | 19.97489175 | 0.000307462 |
| 413 | Unsalted nuts intake \|\| id:ukb-b-12217 |  | rs76520208 | T | C | 0.028099 | 0.0372704 | 0.00777656 | 1.60E-06 | 64949 | 22.96957995 | 0.000353541 |
| 414 | Unsalted nuts intake \|\| id:ukb-b-12217 |  | rs77536627 | T | C | 0.030014 | 0.0356537 | 0.00763562 | 3.00E-06 | 64949 | 21.80321805 | 0.000335595 |
| 415 | Salted nuts intake \|\| id:ukb-b-15960 | 64,949 | rs10845793 | G | C | 0.900684 | -0.0130518 | 0.00272321 | 1.60E-06 | 64949 | 22.97092871 | 0.000353562 |
| 416 | Salted nuts intake \|\| id:ukb-b-15960 |  | rs10996983 | T | C | 0.021882 | 0.0253131 | 0.00552076 | 4.50E-06 | 64949 | 21.02291499 | 0.000323589 |
| 417 | Salted nuts intake \|\| id:ukb-b-15960 |  | rs116089899 | G | C | 0.007766 | 0.0473616 | 0.00948749 | 6.00E-07 | 64949 | 24.92011653 | 0.000383552 |
| 418 | Salted nuts intake \|\| id:ukb-b-15960 |  | rs116968994 | T | C | 0.009407 | 0.0369948 | 0.00834077 | 9.20E-06 | 64949 | 19.67293135 | 0.000302816 |
| 419 | Salted nuts intake \|\| id:ukb-b-15960 |  | rs117345122 | T | G | 0.039794 | 0.0199755 | 0.00414606 | 1.50E-06 | 64949 | 23.21261948 | 0.000357281 |
| 420 | Salted nuts intake \|\| id:ukb-b-15960 |  | rs117416488 | T | C | 0.007475 | 0.0420853 | 0.00933244 | 6.50E-06 | 64949 | 20.33622985 | 0.000313022 |
| 421 | Salted nuts intake \|\| id:ukb-b-15960 |  | rs13284665 | G | A | 0.131761 | 0.0144441 | 0.00237596 | 1.20E-09 | 64949 | 36.95751171 | 0.000568717 |
| 422 | Salted nuts intake \|\| id:ukb-b-15960 |  | rs1381597 | T | C | 0.716245 | -0.00827381 | 0.00178175 | 3.40E-06 | 64949 | 21.56341554 | 0.000331905 |
| 423 | Salted nuts intake \|\| id:ukb-b-15960 |  | rs138188349 | G | A | 0.017319 | 0.0313199 | 0.00635418 | 8.30E-07 | 64949 | 24.29526872 | 0.000373939 |
| 424 | Salted nuts intake \|\| id:ukb-b-15960 |  | rs144425541 | A | G | 0.011905 | 0.0393736 | 0.00814235 | 1.30E-06 | 64949 | 23.38356464 | 0.000359911 |
| 425 | Salted nuts intake \|\| id:ukb-b-15960 |  | rs145621746 | T | G | 0.035585 | 0.0206888 | 0.00454779 | 5.40E-06 | 64949 | 20.6952081 | 0.000318546 |
| 426 | Salted nuts intake \|\| id:ukb-b-15960 |  | rs145771464 | C | G | 0.020571 | 0.0265034 | 0.0058956 | 6.90E-06 | 64949 | 20.2091088 | 0.000311066 |
| 427 | Salted nuts intake \|\| id:ukb-b-15960 |  | rs146665115 | A | G | 0.008046 | 0.0449273 | 0.00971907 | 3.80E-06 | 64949 | 21.36836134 | 0.000328904 |
| 428 | Salted nuts intake \|\| id:ukb-b-15960 |  | rs147958200 | T | C | 0.015339 | 0.0320672 | 0.00687906 | 3.10E-06 | 64949 | 21.73020813 | 0.000334472 |
| 429 | Salted nuts intake \|\| id:ukb-b-15960 |  | rs148012549 | A | T | 0.019552 | 0.0267846 | 0.00597158 | 7.30E-06 | 64949 | 20.11832503 | 0.000309669 |
| 430 | Salted nuts intake \|\| id:ukb-b-15960 |  | rs148448084 | G | A | 0.02969 | 0.0217994 | 0.00479212 | 5.40E-06 | 64949 | 20.69348856 | 0.00031852 |
| 431 | Salted nuts intake \|\| id:ukb-b-15960 |  | rs150856188 | T | G | 0.010508 | 0.0394604 | 0.00804891 | 9.50E-07 | 64949 | 24.03525997 | 0.000369938 |
| 432 | Salted nuts intake \|\| id:ukb-b-15960 |  | rs1653772 | G | A | 0.984534 | -0.0308433 | 0.00650544 | 2.10E-06 | 64949 | 22.47855163 | 0.000345986 |
| 433 | Salted nuts intake \|\| id:ukb-b-15960 |  | rs34252874 | C | T | 0.476176 | 0.00724278 | 0.00163898 | 9.90E-06 | 64949 | 19.52824789 | 0.000300589 |
| 434 | Salted nuts intake \|\| id:ukb-b-15960 |  | rs4972701 | T | C | 0.467924 | -0.00749931 | 0.00161862 | 3.60E-06 | 64949 | 21.4660832 | 0.000330408 |
| 435 | Salted nuts intake \|\| id:ukb-b-15960 |  | rs506454 | C | T | 0.011994 | 0.0360269 | 0.00765093 | 2.50E-06 | 64949 | 22.17304463 | 0.000341286 |
| 436 | Salted nuts intake \|\| id:ukb-b-15960 |  | rs55768166 | G | A | 0.181033 | 0.00962393 | 0.00209658 | 4.40E-06 | 64949 | 21.07084895 | 0.000324326 |
| 437 | Salted nuts intake \|\| id:ukb-b-15960 |  | rs55830729 | C | G | 0.037865 | 0.0201115 | 0.00425077 | 2.20E-06 | 64949 | 22.38482548 | 0.000344544 |
| 438 | Salted nuts intake \|\| id:ukb-b-15960 |  | rs56033844 | T | C | 0.099143 | 0.0119605 | 0.00268651 | 8.50E-06 | 64949 | 19.82082595 | 0.000305092 |
| 439 | Salted nuts intake \|\| id:ukb-b-15960 |  | rs59732881 | C | T | 0.363208 | -0.00744448 | 0.00166722 | 8.00E-06 | 64949 | 19.93806062 | 0.000306895 |
| 440 | Salted nuts intake \|\| id:ukb-b-15960 |  | rs72822396 | C | G | 0.102401 | -0.0117756 | 0.00266014 | 9.60E-06 | 64949 | 19.59553398 | 0.000301625 |
| 441 | Salted nuts intake \|\| id:ukb-b-15960 |  | rs73085383 | T | C | 0.014743 | 0.0372358 | 0.00705217 | 1.30E-07 | 64949 | 27.87891272 | 0.000429072 |
| 442 | Salted nuts intake \|\| id:ukb-b-15960 |  | rs75310568 | T | G | 0.014776 | 0.0349862 | 0.00789886 | 9.50E-06 | 64949 | 19.61845109 | 0.000301977 |
| 443 | Salted nuts intake \|\| id:ukb-b-15960 |  | rs75342409 | C | T | 0.015501 | 0.0354039 | 0.00666377 | 1.10E-07 | 64949 | 28.22683683 | 0.000434425 |
| 444 | Salted nuts intake \|\| id:ukb-b-15960 |  | rs79188173 | A | G | 0.012289 | 0.0348586 | 0.00771822 | 6.30E-06 | 64949 | 20.39790546 | 0.000313971 |
| 445 | Salted nuts intake \|\| id:ukb-b-15960 |  | rs79611807 | G | A | 0.029977 | 0.0213085 | 0.00472025 | 6.40E-06 | 64949 | 20.37866621 | 0.000313675 |
| 446 | Salted nuts intake \|\| id:ukb-b-15960 |  | rs79900875 | G | A | 0.041978 | 0.0190884 | 0.00411566 | 3.50E-06 | 64949 | 21.51097395 | 0.000331098 |
| 447 | Salted nuts intake \|\| id:ukb-b-15960 |  | rs79961252 | T | A | 0.013428 | 0.0333635 | 0.00751822 | 9.10E-06 | 64949 | 19.69305746 | 0.000303125 |
| 448 | Salted nuts intake \|\| id:ukb-b-15960 |  | rs80261113 | T | G | 0.007293 | 0.0537207 | 0.00991227 | 6.00E-08 | 64949 | 29.37224077 | 0.000452045 |
| 449 | Salted nuts intake \|\| id:ukb-b-15960 |  | rs80265037 | T | G | 0.049991 | 0.0171419 | 0.00372793 | 4.30E-06 | 64949 | 21.14376955 | 0.000325448 |
| 450 | Salted nuts intake \|\| id:ukb-b-15960 |  | rs906822 | T | G | 0.067202 | 0.0151811 | 0.00322679 | 2.50E-06 | 64949 | 22.13426358 | 0.000340689 |
| 451 | Salted nuts intake \|\| id:ukb-b-15960 |  | rs9613266 | C | T | 0.468321 | -0.00775525 | 0.00160513 | 1.40E-06 | 64949 | 23.34377997 | 0.000359299 |
| 452 | Salted nuts intake \|\| id:ukb-b-15960 |  | rs9705504 | A | G | 0.844629 | -0.0102161 | 0.00224239 | 5.20E-06 | 64949 | 20.75620611 | 0.000319485 |
| 453 | Lamb intake \|\| id:ukb-b-530 | 64,942 | rs10001236 | T | G | 0.332317 | 0.012621 | 0.00274786 | 4.40E-06 | 64942 | 21.09591316 | 0.000324747 |
| 454 | Lamb intake \|\| id:ukb-b-530 |  | rs1156065 | T | C | 0.497241 | 0.0123646 | 0.00259653 | 1.90E-06 | 64942 | 22.67636575 | 0.000349068 |
| 455 | Lamb intake \|\| id:ukb-b-530 |  | rs116822304 | A | G | 0.055248 | 0.0268795 | 0.0057795 | 3.30E-06 | 64942 | 21.63026478 | 0.00033297 |
| 456 | Lamb intake \|\| id:ukb-b-530 |  | rs11687610 | C | T | 0.317461 | 0.0143342 | 0.00278151 | 2.60E-07 | 64942 | 26.55740693 | 0.000408786 |
| 457 | Lamb intake \|\| id:ukb-b-530 |  | rs116908511 | T | G | 0.036193 | -0.0311325 | 0.00697698 | 8.10E-06 | 64942 | 19.9109984 | 0.000306512 |
| 458 | Lamb intake \|\| id:ukb-b-530 |  | rs12338051 | A | G | 0.122585 | 0.0186968 | 0.00394993 | 2.20E-06 | 64942 | 22.40555811 | 0.0003449 |
| 459 | Lamb intake \|\| id:ukb-b-530 |  | rs12456489 | G | A | 0.283082 | 0.0131279 | 0.00294725 | 8.40E-06 | 64942 | 19.84068066 | 0.00030543 |
| 460 | Lamb intake \|\| id:ukb-b-530 |  | rs12933901 | C | G | 0.35474 | -0.0124185 | 0.00269513 | 4.10E-06 | 64942 | 21.23141047 | 0.000326832 |
| 461 | Lamb intake \|\| id:ukb-b-530 |  | rs1367503 | A | G | 0.057979 | -0.0249174 | 0.00551587 | 6.30E-06 | 64942 | 20.40691728 | 0.000314144 |
| 462 | Lamb intake \|\| id:ukb-b-530 |  | rs140791170 | G | A | 0.011847 | 0.0623054 | 0.0133215 | 2.90E-06 | 64942 | 21.87485177 | 0.000336734 |
| 463 | Lamb intake \|\| id:ukb-b-530 |  | rs142252464 | T | C | 0.009735 | 0.0856969 | 0.0145706 | 4.10E-09 | 64942 | 34.59197452 | 0.000532392 |
| 464 | Lamb intake \|\| id:ukb-b-530 |  | rs146003962 | A | G | 0.015392 | 0.0531839 | 0.0105626 | 4.80E-07 | 64942 | 25.35237763 | 0.000390245 |
| 465 | Lamb intake \|\| id:ukb-b-530 |  | rs147152844 | A | C | 0.020385 | 0.0431184 | 0.00951911 | 5.90E-06 | 64942 | 20.51788462 | 0.000315852 |
| 466 | Lamb intake \|\| id:ukb-b-530 |  | rs147244930 | C | T | 0.046958 | 0.0300596 | 0.00622018 | 1.30E-06 | 64942 | 23.3539577 | 0.000359494 |
| 467 | Lamb intake \|\| id:ukb-b-530 |  | rs148058638 | G | A | 0.00941 | 0.0610977 | 0.0135086 | 6.10E-06 | 64942 | 20.45639209 | 0.000314905 |
| 468 | Lamb intake \|\| id:ukb-b-530 |  | rs150703524 | T | A | 0.011564 | 0.0606624 | 0.0125629 | 1.40E-06 | 64942 | 23.31628586 | 0.000358915 |
| 469 | Lamb intake \|\| id:ukb-b-530 |  | rs150769370 | T | C | 0.02466 | -0.0399515 | 0.00884693 | 6.30E-06 | 64942 | 20.39299429 | 0.00031393 |
| 470 | Lamb intake \|\| id:ukb-b-530 |  | rs17265218 | A | G | 0.186514 | 0.0149162 | 0.00335561 | 8.80E-06 | 64942 | 19.75938557 | 0.000304179 |
| 471 | Lamb intake \|\| id:ukb-b-530 |  | rs17510124 | C | T | 0.028206 | 0.035268 | 0.00779568 | 6.10E-06 | 64942 | 20.46697489 | 0.000315068 |
| 472 | Lamb intake \|\| id:ukb-b-530 |  | rs183332560 | G | T | 0.012644 | 0.0564299 | 0.0125022 | 6.40E-06 | 64942 | 20.37256336 | 0.000313615 |
| 473 | Lamb intake \|\| id:ukb-b-530 |  | rs185833372 | C | A | 0.031901 | 0.0363174 | 0.00761827 | 1.90E-06 | 64942 | 22.72567428 | 0.000349826 |
| 474 | Lamb intake \|\| id:ukb-b-530 |  | rs2041317 | T | G | 0.171356 | 0.0156788 | 0.0034867 | 6.90E-06 | 64942 | 20.22071355 | 0.000311278 |
| 475 | Lamb intake \|\| id:ukb-b-530 |  | rs74380169 | A | G | 0.049608 | 0.0277714 | 0.00595969 | 3.20E-06 | 64942 | 21.71441869 | 0.000334265 |
| 476 | Lamb intake \|\| id:ukb-b-530 |  | rs76037117 | A | G | 0.015914 | 0.0480445 | 0.0102848 | 3.00E-06 | 64942 | 21.82205543 | 0.000335921 |
| 477 | Lamb intake \|\| id:ukb-b-530 |  | rs78938647 | G | A | 0.016356 | 0.04821 | 0.010288 | 2.80E-06 | 64942 | 21.95898954 | 0.000338028 |
| 478 | Indian snacks intake \|\| id:ukb-b-282 | 64,949 | rs10419256 | A | G | 0.130757 | 0.0110489 | 0.00243215 | 5.50E-06 | 64949 | 20.63751366 | 0.000317658 |
| 479 | Indian snacks intake \|\| id:ukb-b-282 |  | rs111778611 | A | G | 0.01396 | 0.0327615 | 0.00723963 | 6.00E-06 | 64949 | 20.47834304 | 0.000315209 |
| 480 | Indian snacks intake \|\| id:ukb-b-282 |  | rs111957747 | A | G | 0.009548 | 0.0392673 | 0.00879691 | 8.10E-06 | 64949 | 19.92515843 | 0.000306697 |
| 481 | Indian snacks intake \|\| id:ukb-b-282 |  | rs112720655 | C | A | 0.019264 | 0.0299482 | 0.00654429 | 4.70E-06 | 64949 | 20.94191426 | 0.000322342 |
| 482 | Indian snacks intake \|\| id:ukb-b-282 |  | rs11595876 | C | T | 0.070851 | 0.014753 | 0.00327499 | 6.60E-06 | 64949 | 20.29274174 | 0.000312353 |
| 483 | Indian snacks intake \|\| id:ukb-b-282 |  | rs11649662 | G | C | 0.062901 | 0.0162677 | 0.00343161 | 2.10E-06 | 64949 | 22.4727624 | 0.000345897 |
| 484 | Indian snacks intake \|\| id:ukb-b-282 |  | rs117355815 | T | G | 0.023834 | 0.0237601 | 0.00537522 | 9.90E-06 | 64949 | 19.53907682 | 0.000300756 |
| 485 | Indian snacks intake \|\| id:ukb-b-282 |  | rs117616173 | C | T | 0.022325 | 0.0261611 | 0.00553383 | 2.30E-06 | 64949 | 22.3491173 | 0.000343995 |
| 486 | Indian snacks intake \|\| id:ukb-b-282 |  | rs117990599 | T | C | 0.027484 | 0.0255735 | 0.00576657 | 9.20E-06 | 64949 | 19.66732086 | 0.000302729 |
| 487 | Indian snacks intake \|\| id:ukb-b-282 |  | rs11872167 | A | G | 0.036621 | 0.0199057 | 0.00436098 | 5.00E-06 | 64949 | 20.83467446 | 0.000320692 |
| 488 | Indian snacks intake \|\| id:ukb-b-282 |  | rs12784972 | A | G | 0.383489 | 0.00808896 | 0.00168162 | 1.50E-06 | 64949 | 23.13820411 | 0.000356136 |
| 489 | Indian snacks intake \|\| id:ukb-b-282 |  | rs138151469 | T | C | 0.005796 | 0.074295 | 0.0113711 | 6.40E-11 | 64949 | 42.6888265 | 0.000656855 |
| 490 | Indian snacks intake \|\| id:ukb-b-282 |  | rs138398208 | G | A | 0.022113 | 0.0267719 | 0.00577823 | 3.60E-06 | 64949 | 21.46687062 | 0.00033042 |
| 491 | Indian snacks intake \|\| id:ukb-b-282 |  | rs138744166 | T | A | 0.007658 | 0.0441458 | 0.00987314 | 7.80E-06 | 64949 | 19.99255006 | 0.000307734 |
| 492 | Indian snacks intake \|\| id:ukb-b-282 |  | rs139579724 | G | A | 0.00984 | 0.043315 | 0.00869829 | 6.40E-07 | 64949 | 24.79755554 | 0.000381666 |
| 493 | Indian snacks intake \|\| id:ukb-b-282 |  | rs140965084 | T | C | 0.01822 | 0.0287846 | 0.00639528 | 6.80E-06 | 64949 | 20.2582194 | 0.000311822 |
| 494 | Indian snacks intake \|\| id:ukb-b-282 |  | rs141364295 | T | C | 0.012208 | 0.0495353 | 0.00882611 | 2.00E-08 | 64949 | 31.49857622 | 0.000484754 |
| 495 | Indian snacks intake \|\| id:ukb-b-282 |  | rs143022692 | A | G | 0.00959 | 0.0420886 | 0.00878476 | 1.70E-06 | 64949 | 22.9545698 | 0.000353311 |
| 496 | Indian snacks intake \|\| id:ukb-b-282 |  | rs143153761 | T | G | 0.009315 | 0.046762 | 0.00928476 | 4.70E-07 | 64949 | 25.36557962 | 0.000390406 |
| 497 | Indian snacks intake \|\| id:ukb-b-282 |  | rs143452328 | A | G | 0.012627 | 0.0373389 | 0.00784832 | 2.00E-06 | 64949 | 22.63443377 | 0.000348385 |
| 498 | Indian snacks intake \|\| id:ukb-b-282 |  | rs144052134 | A | G | 0.009659 | 0.0408327 | 0.00914818 | 8.10E-06 | 64949 | 19.92263569 | 0.000306658 |
| 499 | Indian snacks intake \|\| id:ukb-b-282 |  | rs146057934 | T | C | 0.022438 | 0.0294058 | 0.00581406 | 4.20E-07 | 64949 | 25.58037929 | 0.00039371 |
| 500 | Indian snacks intake \|\| id:ukb-b-282 |  | rs146431609 | A | G | 0.017304 | 0.034241 | 0.00659448 | 2.10E-07 | 64949 | 26.96073753 | 0.000414947 |
| 501 | Indian snacks intake \|\| id:ukb-b-282 |  | rs148210187 | A | G | 0.013318 | 0.0348867 | 0.00726567 | 1.60E-06 | 64949 | 23.0551761 | 0.000354858 |
| 502 | Indian snacks intake \|\| id:ukb-b-282 |  | rs148617199 | C | A | 0.007422 | 0.0447287 | 0.00987177 | 5.90E-06 | 64949 | 20.52969491 | 0.000315999 |
| 503 | Indian snacks intake \|\| id:ukb-b-282 |  | rs148744609 | A | G | 0.011736 | 0.0348076 | 0.00777075 | 7.50E-06 | 64949 | 20.06422017 | 0.000308837 |
| 504 | Indian snacks intake \|\| id:ukb-b-282 |  | rs149588711 | G | T | 0.011356 | 0.0410996 | 0.00891055 | 4.00E-06 | 64949 | 21.27483337 | 0.000327465 |
| 505 | Indian snacks intake \|\| id:ukb-b-282 |  | rs149617817 | C | G | 0.005936 | 0.0534077 | 0.0110708 | 1.40E-06 | 64949 | 23.27285914 | 0.000358208 |
| 506 | Indian snacks intake \|\| id:ukb-b-282 |  | rs149817908 | T | G | 0.010185 | 0.0379842 | 0.00852968 | 8.50E-06 | 64949 | 19.83081171 | 0.000305245 |
| 507 | Indian snacks intake \|\| id:ukb-b-282 |  | rs150604194 | T | C | 0.010443 | 0.0370819 | 0.00825973 | 7.10E-06 | 64949 | 20.15543867 | 0.00031024 |
| 508 | Indian snacks intake \|\| id:ukb-b-282 |  | rs17031476 | G | T | 0.017595 | 0.0276493 | 0.00621842 | 8.70E-06 | 64949 | 19.7700687 | 0.00030431 |
| 509 | Indian snacks intake \|\| id:ukb-b-282 |  | rs1828087 | A | G | 0.105718 | 0.0130783 | 0.00274903 | 2.00E-06 | 64949 | 22.63307862 | 0.000348364 |
| 510 | Indian snacks intake \|\| id:ukb-b-282 |  | rs2242370 | T | A | 0.399177 | 0.00756689 | 0.00167493 | 6.30E-06 | 64949 | 20.40993016 | 0.000314156 |
| 511 | Indian snacks intake \|\| id:ukb-b-282 |  | rs2510706 | C | T | 0.409114 | -0.00750488 | 0.00166778 | 6.80E-06 | 64949 | 20.24929847 | 0.000311685 |
| 512 | Indian snacks intake \|\| id:ukb-b-282 |  | rs35367621 | C | T | 0.010797 | 0.0414769 | 0.00915576 | 5.90E-06 | 64949 | 20.52219355 | 0.000315884 |
| 513 | Indian snacks intake \|\| id:ukb-b-282 |  | rs4343387 | A | G | 0.184849 | 0.00962141 | 0.00210835 | 5.00E-06 | 64949 | 20.82533647 | 0.000320548 |
| 514 | Indian snacks intake \|\| id:ukb-b-282 |  | rs4649395 | T | G | 0.010216 | 0.0377983 | 0.00825939 | 4.70E-06 | 64949 | 20.94346755 | 0.000322366 |
| 515 | Indian snacks intake \|\| id:ukb-b-282 |  | rs4900026 | A | G | 0.704922 | -0.00806672 | 0.00181225 | 8.50E-06 | 64949 | 19.81334257 | 0.000304976 |
| 516 | Indian snacks intake \|\| id:ukb-b-282 |  | rs4916724 | T | C | 0.793097 | -0.00982882 | 0.00202612 | 1.20E-06 | 64949 | 23.53273674 | 0.000362206 |
| 517 | Indian snacks intake \|\| id:ukb-b-282 |  | rs4976673 | T | C | 0.00699 | 0.043873 | 0.00982076 | 7.90E-06 | 64949 | 19.95742328 | 0.000307193 |
| 518 | Indian snacks intake \|\| id:ukb-b-282 |  | rs61966187 | C | A | 0.026328 | 0.0295038 | 0.00510612 | 7.60E-09 | 64949 | 33.38672936 | 0.000513797 |
| 519 | Indian snacks intake \|\| id:ukb-b-282 |  | rs62265578 | C | T | 0.252282 | 0.00842806 | 0.00189151 | 8.40E-06 | 64949 | 19.85353995 | 0.000305595 |
| 520 | Indian snacks intake \|\| id:ukb-b-282 |  | rs62483673 | A | C | 0.264792 | 0.00827332 | 0.00186734 | 9.40E-06 | 64949 | 19.62966304 | 0.00030215 |
| 521 | Indian snacks intake \|\| id:ukb-b-282 |  | rs680086 | T | C | 0.009733 | 0.0421587 | 0.00887897 | 2.10E-06 | 64949 | 22.54494831 | 0.000347008 |
| 522 | Indian snacks intake \|\| id:ukb-b-282 |  | rs6803697 | A | G | 0.312087 | 0.00793596 | 0.00177593 | 7.90E-06 | 64949 | 19.96858324 | 0.000307365 |
| 523 | Indian snacks intake \|\| id:ukb-b-282 |  | rs6963707 | A | C | 0.20028 | -0.00953498 | 0.00210907 | 6.20E-06 | 64949 | 20.43890309 | 0.000314602 |
| 524 | Indian snacks intake \|\| id:ukb-b-282 |  | rs72696787 | T | C | 0.101687 | 0.0139978 | 0.00270637 | 2.30E-07 | 64949 | 26.75132063 | 0.000411725 |
| 525 | Indian snacks intake \|\| id:ukb-b-282 |  | rs7274536 | A | G | 0.307028 | -0.00798292 | 0.00177627 | 7.00E-06 | 64949 | 20.19787091 | 0.000310893 |
| 526 | Indian snacks intake \|\| id:ukb-b-282 |  | rs727971 | C | T | 0.047961 | 0.0182309 | 0.00381881 | 1.80E-06 | 64949 | 22.79082863 | 0.000350791 |
| 527 | Indian snacks intake \|\| id:ukb-b-282 |  | rs73994640 | G | C | 0.039252 | -0.0209959 | 0.00421243 | 6.20E-07 | 64949 | 24.8429747 | 0.000382365 |
| 528 | Indian snacks intake \|\| id:ukb-b-282 |  | rs74379938 | C | A | 0.020561 | 0.0263686 | 0.00578762 | 5.20E-06 | 64949 | 20.75745604 | 0.000319504 |
| 529 | Indian snacks intake \|\| id:ukb-b-282 |  | rs74512616 | T | C | 0.021917 | 0.0264366 | 0.00587568 | 6.80E-06 | 64949 | 20.24393477 | 0.000311602 |
| 530 | Indian snacks intake \|\| id:ukb-b-282 |  | rs74881507 | T | C | 0.045474 | 0.0176059 | 0.00393087 | 7.50E-06 | 64949 | 20.06037739 | 0.000308778 |
| 531 | Indian snacks intake \|\| id:ukb-b-282 |  | rs76316510 | T | C | 0.011169 | 0.0361905 | 0.00785324 | 4.10E-06 | 64949 | 21.23691489 | 0.000326882 |
| 532 | Indian snacks intake \|\| id:ukb-b-282 |  | rs7693123 | C | G | 0.973594 | -0.022819 | 0.00511515 | 8.20E-06 | 64949 | 19.90107192 | 0.000306326 |
| 533 | Indian snacks intake \|\| id:ukb-b-282 |  | rs8067112 | G | A | 0.009771 | 0.0368533 | 0.00834012 | 9.90E-06 | 64949 | 19.52576986 | 0.000300551 |
| 534 | Watercress intake \|\| id:ukb-b-6181 | 64,949 | rs111342415 | A | G | 0.067483 | 0.0245548 | 0.00523575 | 2.70E-06 | 64949 | 21.99454588 | 0.000338539 |
| 535 | Watercress intake \|\| id:ukb-b-6181 |  | rs114107092 | A | G | 0.010535 | 0.0702288 | 0.0151309 | 3.50E-06 | 64949 | 21.54274165 | 0.000331587 |
| 536 | Watercress intake \|\| id:ukb-b-6181 |  | rs115479010 | G | C | 0.012968 | 0.0542835 | 0.0116215 | 3.00E-06 | 64949 | 21.81781777 | 0.00033582 |
| 537 | Watercress intake \|\| id:ukb-b-6181 |  | rs116023894 | A | G | 0.02623 | 0.0373581 | 0.00811845 | 4.20E-06 | 64949 | 21.17499517 | 0.000325929 |
| 538 | Watercress intake \|\| id:ukb-b-6181 |  | rs11698271 | T | C | 0.190709 | 0.0147897 | 0.00325959 | 5.70E-06 | 64949 | 20.58698631 | 0.000316881 |
| 539 | Watercress intake \|\| id:ukb-b-6181 |  | rs12141896 | T | C | 0.051707 | 0.0311056 | 0.00614894 | 4.20E-07 | 64949 | 25.5903753 | 0.000393864 |
| 540 | Watercress intake \|\| id:ukb-b-6181 |  | rs143291156 | T | C | 0.019449 | 0.0419578 | 0.00937132 | 7.60E-06 | 64949 | 20.04582253 | 0.000308554 |
| 541 | Watercress intake \|\| id:ukb-b-6181 |  | rs144170411 | T | C | 0.010435 | 0.0574459 | 0.0129844 | 9.70E-06 | 64949 | 19.57376202 | 0.00030129 |
| 542 | Watercress intake \|\| id:ukb-b-6181 |  | rs1447897 | G | A | 0.614754 | -0.0124267 | 0.00264651 | 2.70E-06 | 64949 | 22.04776388 | 0.000339358 |
| 543 | Watercress intake \|\| id:ukb-b-6181 |  | rs147212631 | A | C | 0.021919 | 0.047257 | 0.0103931 | 5.40E-06 | 64949 | 20.67483651 | 0.000318233 |
| 544 | Watercress intake \|\| id:ukb-b-6181 |  | rs149389634 | T | C | 0.011998 | 0.0543197 | 0.0120734 | 6.80E-06 | 64949 | 20.2420991 | 0.000311574 |
| 545 | Watercress intake \|\| id:ukb-b-6181 |  | rs187693683 | G | T | 0.011943 | 0.0582808 | 0.0118037 | 7.90E-07 | 64949 | 24.37893239 | 0.000375226 |
| 546 | Watercress intake \|\| id:ukb-b-6181 |  | rs191852210 | G | A | 0.008862 | 0.0684046 | 0.0145894 | 2.80E-06 | 64949 | 21.98344505 | 0.000338368 |
| 547 | Watercress intake \|\| id:ukb-b-6181 |  | rs3732864 | C | A | 0.525068 | -0.0125512 | 0.00276909 | 5.80E-06 | 64949 | 20.54453696 | 0.000316228 |
| 548 | Watercress intake \|\| id:ukb-b-6181 |  | rs504383 | G | T | 0.340104 | -0.012016 | 0.00269823 | 8.50E-06 | 64949 | 19.83178959 | 0.00030526 |
| 549 | Watercress intake \|\| id:ukb-b-6181 |  | rs55718637 | C | A | 0.0493 | -0.0278627 | 0.00590553 | 2.40E-06 | 64949 | 22.26017825 | 0.000342626 |
| 550 | Watercress intake \|\| id:ukb-b-6181 |  | rs61917839 | C | T | 0.256124 | -0.0140381 | 0.00294505 | 1.90E-06 | 64949 | 22.72120283 | 0.00034972 |
| 551 | Watercress intake \|\| id:ukb-b-6181 |  | rs7080560 | G | A | 0.102595 | 0.0194428 | 0.00431847 | 6.70E-06 | 64949 | 20.27018926 | 0.000312006 |
| 552 | Watercress intake \|\| id:ukb-b-6181 |  | rs72695269 | T | C | 0.036356 | 0.0324203 | 0.00711077 | 5.10E-06 | 64949 | 20.7874298 | 0.000319965 |
| 553 | Watercress intake \|\| id:ukb-b-6181 |  | rs75359554 | C | T | 0.006815 | 0.0726552 | 0.0161389 | 6.70E-06 | 64949 | 20.2668169 | 0.000311954 |
| 554 | Watercress intake \|\| id:ukb-b-6181 |  | rs76766643 | G | A | 0.017668 | 0.0436669 | 0.00970431 | 6.80E-06 | 64949 | 20.24768615 | 0.00031166 |
| 555 | Watercress intake \|\| id:ukb-b-6181 |  | rs76868681 | C | T | 0.008785 | 0.0635584 | 0.0137112 | 3.60E-06 | 64949 | 21.48794977 | 0.000330744 |
| 556 | Watercress intake \|\| id:ukb-b-6181 |  | rs7704452 | G | A | 0.206475 | 0.0147649 | 0.00316795 | 3.20E-06 | 64949 | 21.72222875 | 0.000334349 |
| 557 | Watercress intake \|\| id:ukb-b-6181 |  | rs77102708 | A | G | 0.009069 | 0.060727 | 0.0134072 | 5.90E-06 | 64949 | 20.51575376 | 0.000315785 |
